# Concomitant western diet and chronic-binge alcohol dysregulate hepatic metabolism

**DOI:** 10.1371/journal.pone.0281954

**Published:** 2023-05-03

**Authors:** Delfin Gerard Buyco, Joseph L. Dempsey, Eleonora Scorletti, Sookyoung Jeon, Chelsea Lin, Julia Harkin, Susovon Bayen, Emma E. Furth, Jasmin Martin, Monique Delima, Royce Hooks, Jaimarie Sostre-Colón, Sina A. Gharib, Paul M. Titchenell, Rotonya M. Carr

**Affiliations:** 1 Division of Gastroenterology, Department of Medicine, Perelman School of Medicine, University of Pennsylvania, Philadelphia, Pennsylvania, United States of America; 2 Division of Gastroenterology, Department of Medicine, School of Medicine, University of Washington, Seattle, Washington, United States of America; 3 Department of Food Science and Nutrition, Hallym University, Chuncheon, Gangwon-do, Republic of Korea; 4 Department of Pathology and Laboratory Medicine, Perelman School of Medicine, University of Pennsylvania, Philadelphia, Pennsylvania, United States of America; 5 Institute for Diabetes, Obesity, and Metabolism, Perelman School of Medicine, University of Pennsylvania, Philadelphia, Pennsylvania, United States of America; 6 Division of Pulmonary, Critical Care, and Sleep Medicine, Department of Medicine, University of Washington, Seattle, Washington, United States of America; 7 Center for Lung Biology, University of Washington, Seattle, Washington, United States of America; 8 Department of Physiology, Perelman School of Medicine, University of Pennsylvania, Philadelphia, Pennsylvania, United States of America; University of Illinois at Chicago, UNITED STATES

## Abstract

**Background and aims:**

There is significant overlap between non-alcoholic fatty liver disease (NAFLD) and alcohol-associated liver disease (ALD) with regards to risk factors and disease progression. However, the mechanism by which fatty liver disease arises from concomitant obesity and overconsumption of alcohol (syndrome of metabolic and alcohol-associated fatty liver disease; SMAFLD), is not fully understood.

**Methods:**

Male C57BL6/J mice were fed chow diet (Chow) or high-fructose, high-fat, high-cholesterol diet (FFC) for 4 weeks, then administered either saline or ethanol (EtOH, 5% in drinking water) for another 12 weeks. The EtOH treatment also consisted of a weekly 2.5 g EtOH/kg body weight gavage. Markers for lipid regulation, oxidative stress, inflammation, and fibrosis were measured by RT-qPCR, RNA-seq, Western blot, and metabolomics.

**Results:**

Combined FFC-EtOH induced more body weight gain, glucose intolerance, steatosis, and hepatomegaly compared to Chow, EtOH, or FFC. Glucose intolerance by FFC-EtOH was associated with decreased hepatic protein kinase B (AKT) protein expression and increased gluconeogenic gene expression. FFC-EtOH increased hepatic triglyceride and ceramide levels, plasma leptin levels, hepatic Perilipin 2 protein expression, and decreased lipolytic gene expression. FFC and FFC-EtOH also increased AMP-activated protein kinase (AMPK) activation. Finally, FFC-EtOH enriched the hepatic transcriptome for genes involved in immune response and lipid metabolism.

**Conclusions:**

In our model of early SMAFLD, we observed that the combination of an obesogenic diet and alcohol caused more weight gain, promoted glucose intolerance, and contributed to steatosis by dysregulating leptin/AMPK signaling. Our model demonstrates that the combination of an obesogenic diet with a chronic-binge pattern alcohol intake is worse than either insult alone.

## Introduction

Non-alcoholic fatty liver disease (NAFLD) and alcohol-associated liver disease (ALD) are the most common causes of liver injury [1]. NAFLD has been defined by excessive steatosis, or hepatic fat accumulation, without a history of heavy alcohol consumption [2]. Like NAFLD, ALD begins with hepatic steatosis, and although NAFLD and ALD are often identified as distinct conditions, there exists significant overlap between NAFLD and ALD with regards to risk factors and disease progression [3]. For example, both NAFLD and ALD pathogenesis include steatosis, inflammation, intestinal dysbiosis, fibrosis, and cirrhosis [4]. In addition, the metabolic syndrome (MetS) (the constellation of obesity, insulin resistance (IR), dyslipidemia, and hypertension) contributes to disease severity in both NAFLD and ALD [5, 6]. Rather than two separate conditions, fatty liver disease is increasingly viewed as a spectrum between metabolic dysfunction and alcohol-induced liver injury [7, 8]. Thus, our group previously proposed the term “syndrome of metabolic and alcoholic steatohepatitis” to describe severe liver disease in patients with MetS and a history of heavy alcohol consumption [9].

Here, we use the terminology “syndrome of metabolic and alcohol-associated fatty liver disease” (SMAFLD) to describe our experimental model of fatty liver disease that derives from concomitant obesogenic diet and alcohol overconsumption. Like NAFLD and ALD, SMAFLD progresses from steatosis to advanced stages of disease. Previous experimental models of SMAFLD have largely focused on the ensuing inflammatory stage and have demonstrated that concomitant obesogenic diet and alcohol can synergistically dysregulate lipid metabolism, induce oxidative stress, and promote a pro-inflammatory immune response [10]. Limitations of these models include the lack of replication of the dietary or alcohol consumption patterns of humans who develop liver injury and lack of information on the physiologic and metabolic changes that occur at early stages of disease, prior to the development of advanced injury. For example, existing models employ either chronic or binge models of administering alcohol, but the most deleterious drinking pattern for development of ALD is that of chronic alcohol consumption combined with heavy binge drinking [11, 12]. This chronic-binge drinking pattern is recapitulated in the NIAAA model, which increases ALT/AST levels and inflammation in mice [13]. Most existing studies combining obesogenic diet and alcohol also utilize a high-fat diet (HFD), but dietary risk factors for MetS also include foods with high glycemic index and fructose [14]. Indeed, the synergistic role of fats and carbohydrates in NAFLD was demonstrated in a mouse model in which high-fat, high-cholesterol, high-sugar diet induced steatosis in the short term, and steatohepatitis and fibrosis in the long term [15].

Increasingly, steatosis is being recognized as a disease state during which the accumulation of lipotoxic lipid mediators incite ER stress and a subsequent inflammatory cascade of events [16]. Our own temporal examination of the factors that promote liver injury in experimental ALD established that steatosis is marked by the accumulation of lipotoxic lipids and development of insulin resistance prior to the onset of inflammation [17–19]. Therefore, to further unravel SMAFLD pathogenesis, the steatotic phase must be systematically examined. Here, we describe a mouse model that recapitulates the early, steatotic phase of SMAFLD by combining both chronic-binge EtOH consumption and high-fat, high-fructose, high-cholesterol diet and examining its effect on hepatic lipid dysregulation and glucose intolerance—two established risk factors for disease progression in both NAFLD and ALD.

## Methods

### Animal model

**Fig 1:**
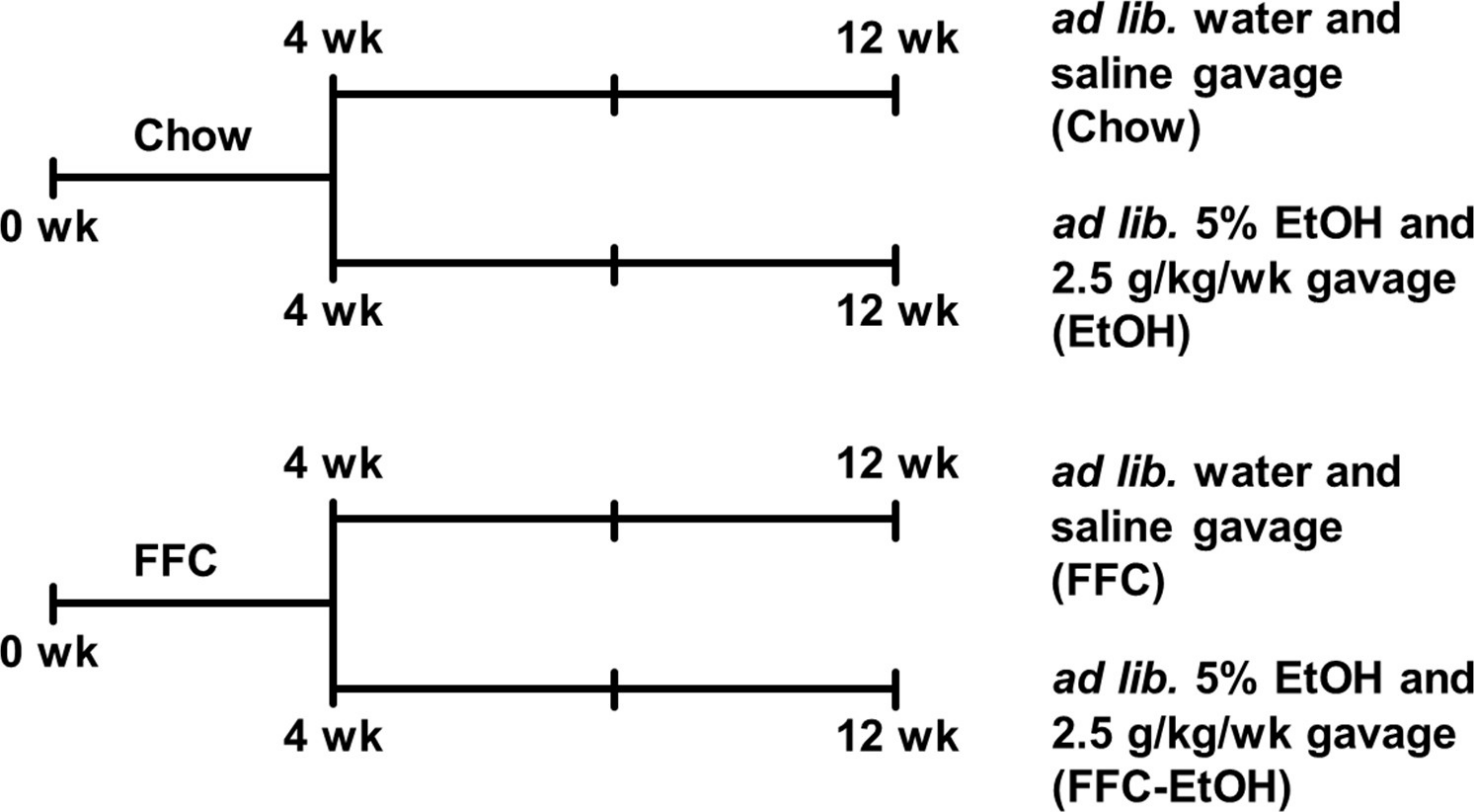
Diagram for the experimental timeline for the present study. A diagram describing the experimental design timeline for the present study. Briefly, eight-week-old male C57BL/6J mice were given ad libitum access to either high fructose, high fat, high cholesterol diet (FFC) or control chow diet. After 4 weeks, half of the mice in each diet group were given ad libitum plain water or 5% EtOH in drinking water and administered 10 mL phosphate-buffered saline (PBS)/kg body weight or 10 mL 31.5% EtOH PBS/kg body weight every week by gavage. This resulted in four treatment groups (n = 5 in each group): chow and saline (Chow), chow and ethanol (EtOH), FFC and saline (FFC), and FFC and EtOH (FFC-EtOH).

Animal experiments were performed with humane care in accordance with the protocols approved by the Institutional Animal Care and Use Committee of the University of Pennsylvania. Eight-week-old male C57BL/6J mice (24.0 ± 0.16 g) were given *ad libitum* access to either high fructose, high fat, high cholesterol diet (FFC; 40 kcal% fat, 20 kcal% fructose, 2% cholesterol; D09100310i, Research Diets, Inc.) or control chow diet. Male mice were chosen to establish this model because of their propensity for insulin resistance and obesity [20] and based on our prior studies demonstrating the onset of steatosis and insulin resistance in 4 weeks with chronic alcohol intake in these mice [19]. After 4 weeks, half of the mice in each diet group were given *ad libitum* 5% EtOH in drinking water and administered 10 mL 31.5% EtOH in phosphate-buffered saline (PBS)/kg body weight every week by gavage (i.e., 2.5 g EtOH/kg body weight). A daily exposure of 2.5 g EtOH/kg body weight was used in other studies [21–23], and higher gavage exposure results in high mortality. The remaining mice were given *ad libitum* plain drinking water and administered 10 mL PBS/kg body weight every week by gavage. Following gavage, the mice were placed under a heat lamp and supervised for one hour. Thus, four treatment groups were created (Fig 1; *n* = 5 in each group): chow and saline (“Chow”), chow and ethanol (“EtOH”), FFC and saline (“FFC”), and FFC and EtOH (“FFC-EtOH”). Eight weeks after the beginning of saline and EtOH treatment, the mice were euthanized, and blood, liver, epididymal white adipose tissue (WAT), and skeletal muscle tissue samples were collected.

### Histology

Formalin-fixed liver tissue samples were embedded in paraffin, cut into sections, and stained with either hematoxylin and eosin stain (H&E) or trichrome stain. Frozen OCT-embedded liver tissues were stained with Oil Red O (ORO) stain. The stained sections were examined using a Nikon Eclipse E600 fluorescence microscope and Nikon NIS-Elements software. Paraffin-embedding, sectioning, and staining were done by the Molecular Pathology and Imaging Core at the University of Pennsylvania.

### Biochemical assays

Plasma samples for biochemical assays were obtained by centrifuging blood in a lithium heparin tube at 2000 × g at 4˚C for 10 min. Liver samples were prepared by adding 6 μL 2:1 EtOH:30% KOH solution per mg of liver tissue, vortexing, then incubating in a 60˚C water bath overnight. Then, 1.08 volumes of 1 MgCl_2_ was added to the liver digest before it was vortexed to a milky consistency, and placed on ice for 10 min. The liver digest was centrifuged at 18000 × g at room temperature for 30 min, and the resulting supernatant was used for biochemical assays.

Plasma alanine aminotransferase (ALT) activity was measured using a Stanbio ALT/SGPT Liqui-UV Test kit (EKF Diagnostics, Texas). Plasma and liver triglyceride (TG) levels were measured using a Stanbio Triglycerides LiquiColor Test Mono (EKF Diagnostics, Texas). Non-esterified fatty acid (NEFA) levels in plasma were measured using a Wako HR Series NEFA-HR 2 test kit (Fujifilm, Japan). All biochemical assays were analyzed use Infinite 200 PRO plate reader (Tecan Trading AG, Switzerland). Plasma insulin, leptin, and adiponectin levels were determined by ELISA by the Radioimmunoassay and Biomarkers Core at Institute for Diabetes, Obesity and Metabolism at the University of Pennsylvania.

### Western blot

Tissue protein lysates were prepared by homogenizing liver, adipose, or muscle tissue in RIPA buffer (50 mM Tris (pH 8.0), 150 mM NaCl, 5 mM EDTA, 1% v/v NP-40, 0.5% w/v sodium deoxycholate, 0.1% v/v SDS, 50 mM sodium fluoride) containing cOmplete protease inhibitor and PhosStop phosphatase inhibitor tablets (Roche Diagnostics, Germany). For adipose tissue, lipids were separated from protein by centrifugation. Protein concentrations were determined using a Pierce BCA protein assay kit (Thermo-Fisher Scientific). Proteins were then resolved on a NuPAGE 4–12% Bis-Tris gel (Invitrogen, CA) by electrophoresis, and transferred onto a nitrocellulose membrane by electroblotting. Blots were probed using primary antibodies listed in S1 Table, and horseradish peroxidase-conjugated secondary antibodies for mouse and rabbit protein. Blots were analyzed using ImageJ software (NIH). Data were normalized to glyceraldehyde 3-phosphate dehydrogenase (GAPDH) protein levels and expressed as fold change from Chow control.

### Quantitative PCR

Liver, adipose, and muscle tissue samples stored in RNAlater solution (Invitrogen, Lithuania) were solubilized using a Bio-Gen PRO 200 homogenizer (PRO Scientific, CT). mRNA was extracted from the homogenate using a PureLINK RNA Mini Kit (Invitrogen, CA). The mRNA samples were treated with Amplification Grade DNase I (Invitrogen, CA), then MultiScribe reverse transcriptase (RT; Applied Biosystems, CA) to produce cDNA. Quantitative real-time PCR (qPCR) was performed with the cDNA on a StepOnePlus PCR system (Applied Biosystems, CA) or Taqman 7900 (Thermo-Fisher Scientific) using gene-specific primers (S1 Table) and SYBR Select Master Mix (Applied Biosystems, Lithuania). RT-qPCR data were normalized to mRNA levels of ribosomal protein, large, P0 (*36b4*) reference gene and expressed as fold change from Chow control.

### Glucose tolerance test

For glucose tolerance tests (GTT), mice were fasted for six hours, then intraperitoneally administered 20% glucose in PBS at a dosage of 10 mL/kg body weight. Blood glucose levels were measured at 0, 15, 30, 60, 120, and 180 min time points with an ACCU-CHEK Inform II glucometer (Roche Diabetes Care, Indiana).

### Ceramide analysis

For hepatic metabolomics analyses, liver tissue was first homogenized in RIPA buffer and protein concentrations were determined by BCA assay. Liver homogenate and plasma samples were analyzed by high performance liquid chromatography-tandem mass spectrometry for ceramide content at the Lipidomics Shared Resource at the Medical University of South Carolina. Hepatic ceramide data were normalized to total protein levels, as measured using a Pierce BCA Protein Assay Kit (Thermo Fisher Scientific).

### RNAseq

Samples for RNAseq analysis were prepared from liver tissue homogenates using a PureLINK RNA Mini Kit (Invitrogen, CA). Libraries were prepared by the Next Generation Sequencing Core at the University of Pennsylvania. Briefly, the libraries where prepared with the TruSeq Stranded mRNA HT Sample Prep Kit (Illumina) kit. RNA and library quality was determined by with an Agilent bioAnalyzer. Sequencing was performed using an Illumina NovaSeq 6000 100bp single-end reads. The Molecular Profiling Facility at the University of Pennsylvania performed data analysis. Raw sequence files (fastq) were mapped using salmon (https://combine-lab.github.io/salmon/) against the mouse transcriptome described in GENCODE vM27. Transcript count data was summarized at the gene level with tximeta (https://bioconductor.org/packages/release/bioc/html/tximeta.html) and annotated with biomaRt. Count data is available in S1 Table. Transcript normalization and statistical analysis was performed with DESeq2 (https://bioconductor.org/packages/release/bioc/html/DESeq2.html) using the Benjamini-Hochberg false discovery rate (BH-FDR). DESeq2 was also used for principle components analysis. The Venn diagram and normalized expression heatmap were created using the R packages VennDiagram and ComplexHeatmap, respectively. Pathway analysis was performed using Gene Set Enrichment Analysis (GSEA) v4.2.3 using the curated gene sets for hallmark genes (h.all.v7.5.1.symbols) and canonical pathways (9c2.cp.v7.5.1.symbols). Pathway analysis visualizations were made using Cytoscape v3.9.1.

### Data analysis

Data analyses were performed using GraphPad Prism 8 (GraphPad Software, Inc., CA) statistical software. Continuous parametric variables were analyzed by One-way ANOVA and Šidák’s multiple comparisons test if the difference in a variable was statistically significant. Continuous non-parametric variables were analyzed by Kruskal-Wallis and Dunn’s test if the difference in a variable was statistically significant. Data were expressed as mean ± SEM. P-values less than 0.05 were considered statistically significant.

## Results

### Alcohol and obesogenic diet synergistically induce weight gain and liver injury

Obesity is observed in both NAFLD and ALD patients and is a feature of SMAFLD [24, 25]. Thus, body weight was measured in mice to determine the role of diet and alcohol in promoting obesity [26, 27]. After 12 weeks, FFC-EtOH significantly increased average body weight (35.9 ± 1.8 g) (Table 1) compared to FFC (30.3 ± 0.8 g; P = 0.01), EtOH (28.2 ± 0.7 g; P < 0.0001), and Chow (27.2 ± 0.2 g; P < 0.0001). Compared to Chow, FFC alone also increased body weight (P = 0.01), but EtOH alone did not. FFC-EtOH also significantly increased liver mass (P = 0.03) and WAT mass (P = 0.04) compared to Chow diet. FFC also induced hepatomegaly and central obesity compared to Chow, however, these changes did not reach statistical significance (Table 1). Finally, there were no signs of tumor development or carcinoma, as is expected for early-stage NAFLD and ALD.

**Table 1:** Body metrics and biochemistry.

	Chow	EtOH	FFC	FFC-EtOH
Body weight (g)	27.2 ± 0.2	28.2 ± 0.7	30.3 ± 0.8^aa^	35.9 ± 1.8^aaabbcc^
Liver mass (g)	1.35 ± 0.03	1.44 ± 0.06	1.52 ± 0.047	1.85 ± 0.20^a^
WAT mass (g)	0.49 ± 0.05	0.45 ± 0.03	0.68 ± 0.20	1.18 ± 0.24^ab^
ALT activity (U/L)	9.60 ± 2.03	11.45 ± 1.21	16.71 ± 3.59	22.18 ± 4.40^a^
NAFLD activity score	0 ± 0	0 ± 0	2 ± 1^a^	3 ± 0^aabb^
Hepatic TG (mg/mL)	13.60 ± 0.80	9.58 ± 1.34	87.15 ± 10.79^aa^	133.04 ± 22.35^aaaabb^
Plasma TG (mg/mL)	86.92 ± 9.35	54.85 ± 4.55^a^	54.79 ± 9.07^a^	54.52 ± 9.03^a^
Plasma NEFA (mEq/L)	0.78 ± 0.08	0.44 ± 0.02^a^	1.04 ± 0.11	0.86 ± 0.06^bb^

Biomarker characterization of whole body and organ mass and lipid concentrations. Significance was determined by one-way ANOVA followed by Kruskal-Wallis and Dunn’s test (compared to Chow: a P ≤ 0.05; aa P ≤ 0.01; aaa P ≤ 0.001; aaaa P ≤ 0.0001; compared to EtOH: b P ≤ 0.05; bb P ≤ 0.01; bbb P ≤ 0.001; bbbb P ≤ 0.0001; compared to FFC: c P ≤ 0.05; cc P ≤ 0.01; ccc P ≤ 0.001; cccc P ≤ 0.0001). Data expressed as mean ± SEM.

**Fig 2:**
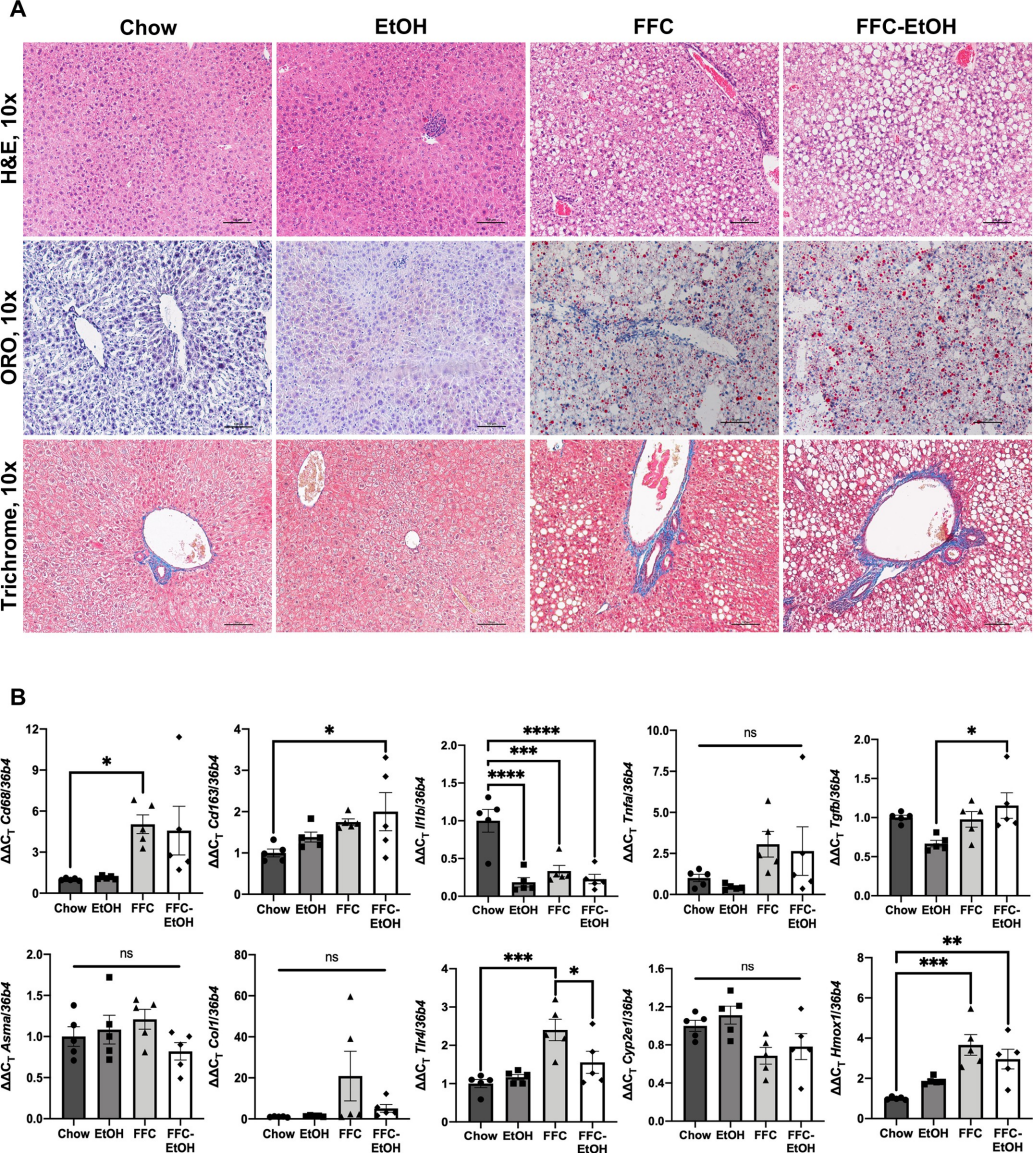
Concomitant obesogenic diet and alcohol induces steatotic liver. (A) Liver histology to determine the phenotypic effect of concomitant obesogenic diet and alcohol. (B) mRNA gene expression by RT-qPCR for pro-inflammatory, pro-fibrotic, and oxidative stress markers. Significance was determined by one-way ANOVA followed by Kruskal-Wallis and Dunn’s test (* p ≤ 0.05; ** p ≤ 0.01; *** p ≤ 0.001; **** p ≤ 0.0001; n = 5/group).

Concomitant obesogenic diet and EtOH consumption synergistically induce liver injury in humans [5]. In our model, while EtOH and FFC tended to raise plasma ALT compared to Chow, only FFC-EtOH significantly increased ALT compared to Chow (P = 0.03) (Table 1). Liver sections were analyzed to determine the effect of concomitant obesogenic diet and alcohol on liver histology (Fig 2A). Both FFC and FFC-EtOH diets induced steatosis compared to Chow on H&E and ORO staining. There was no evidence of steatohepatitis or fibrosis. Consistent with histologic assessment, qualitative steatosis scores demonstrated higher scores compared to Chow for both FFC and FFC-EtOH diets (P = 0.04 and 0.002, respectively); and a trend toward a higher score for FFC-EtOH compared with FFC alone (Table 1). EtOH alone did not induce steatosis in this model, indicating that dietary co-factors are needed to induce more severe ALD [28, 29]. In summary, 12 weeks of FFC-EtOH diet promotes obesity and steatotic liver injury, thus modeling early-stage disease in those patients who develop liver injury resulting from concomitant obesogenic diet and alcohol overconsumption.

### Obesogenic diet drives immune response, oxidative stress, and downregulation in cholesterol synthesis

Despite the lack of discernible inflammation or fibrosis by FFC or EtOH on liver histology, the elevated ALT observed with the combined FFC-EtOH diet suggested worse liver injury than with either insult alone. Thus, we examined pro-inflammatory and pro-fibrotic gene expression to determine the underlying mechanism for the observed liver injury (Fig 2B). FFC upregulated the macrophage marker *Cd68* compared to Chow (P = 0.03), while only FFC-EtOH increased the macrophage-specific protein *Cd163* compared to Chow (P = 0.03). However, FFC-EtOH (P < 0.0001), EtOH (P < 0.0001), and FFC (P = 0.0004) significantly downregulated interleukin 1 beta (*Il1b*) gene expression compared to Chow, while there was no significant difference in tumor necrosis factor alpha (*Tnfa*) between the diet groups. There was also no significant difference in hepatic gene expression of pro-fibrotic genes collagen I (*Col1*) or alpha smooth muscle actin (*Asma*) among the diet groups. Notably, FFC-EtOH increased transforming growth factor beta (*Tgfb*) expression compared to EtOH, suggesting that FFC-EtOH promotes more pro-fibrotic signaling compared to EtOH, as TGFβ induces hepatic stellate cells to produce extracellular matrix proteins such as collagens [30]. Although TGFβ is associated with AKT activation in stellate cell models [31], TGFβ is also known to induce SMAD3-mediated gluconeogenic gene expression with associated decreased AKT activation in mice [32].

Next, we determined whether oxidative stress was implicated in the immune response gene expression profile as suggested by the increase in macrophage markers by FFC and FFC-EtOH (Fig 2B). In a genetic model of obesity, EtOH upregulated oxidative stress markers, such as heme oxygenase 1 (*Hmox1*) [33], while EtOH has also been found to downregulate oxidative stress in HFD-fed mice [34]. In the present model, compared with Chow, FFC-EtOH and FFC significantly upregulated hepatic gene expression of *Hmox1* (P = 0.004 and P = 0.0002, respectively), but there was no significant difference in *Cyp2e1* gene expression between the diet groups. These results indicate that immune response and oxidative stress in this model are primarily driven by FFC, although the increased *Cd163* by FFC-EtOH suggests there may be a synergistic interaction between diet and alcohol.

### Alcohol and obesogenic diet drive hepatic lipid dysregulation

SMAFLD is characterized by lipid dysregulation, as both obesity and alcohol consumption contribute to lipid metabolism dysregulation [35–37]. We therefore measured hepatic and plasma lipid levels (Table 1). FFC increased hepatic TG compared to Chow (P = 0.003), but there was no difference between FFC and FFC-EtOH. Unexpectedly, there was lower plasma TG in the EtOH, FFC, and FFC-EtOH groups compared to Chow (P = 0.04 for all comparisons), but there was no difference between EtOH, FFC, or FFC-EtOH. These results suggest increased *de novo* lipogenesis with TG flux shifted towards storage in hepatic lipid droplets instead of VLDL secretion during the early stage of injury. EtOH also decreased plasma NEFA levels compared to Chow (P = 0.03), while FFC-EtOH increased plasma NEFA compared to EtOH (P = 0.005).

**Fig 3:**
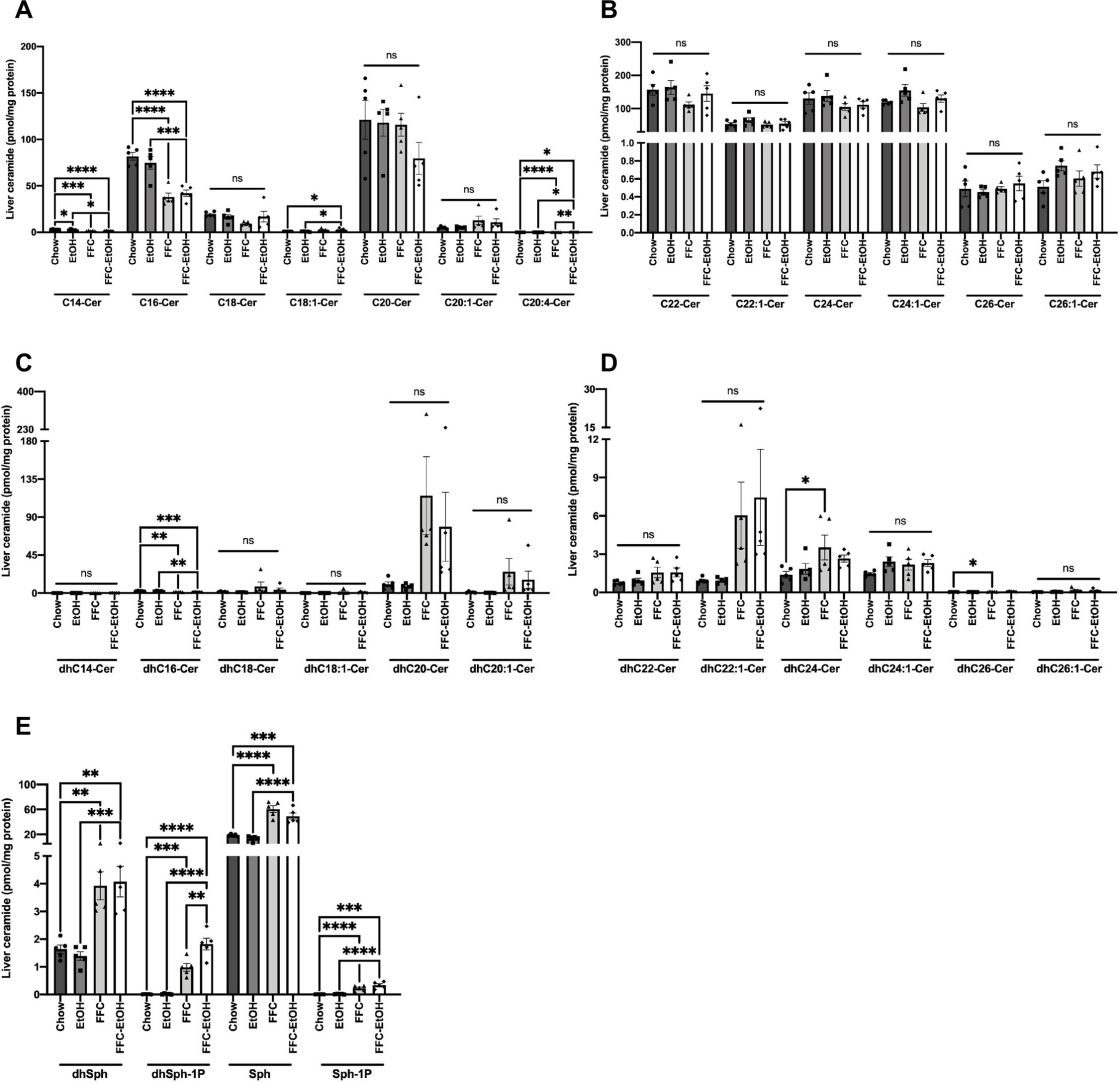
FFC and FFC EtOH increase hepatic long-chain and very long chain ceramides. Hepatic metabolomics for the concentration of (A) long-chain ceramides, (B) very long-chain ceramides, (C) long-chain dihydroceramides, (D) very long-chain dihydroceramides, (E) and sphingosines. Significance was determined by one-way ANOVA followed by Kruskal-Wallis and Dunn’s test (* p ≤ 0.05; ** p ≤ 0.01; *** p ≤ 0.001; **** p ≤ 0.0001; n = 5/group).

Because ceramides are implicated in insulin resistance and inflammation in NAFLD and ALD [38] and we previously demonstrated that ceramides increase concomitant with the onset of steatosis [19], we analyzed hepatic ceramide (Cer) and sphingosine levels. FFC increased hepatic long-chain Cer (Fig 3A) while EtOH tended to increase hepatic very long-chain Cer (Fig 3B). FFC also increased hepatic long-chain dihydroceramides (dhCer) (Fig 3C). However, FFC-EtOH did not significantly affect hepatic Cer content compared to FFC alone. FFC also increased hepatic sphingosines (Fig 3E).

**Fig 4:**
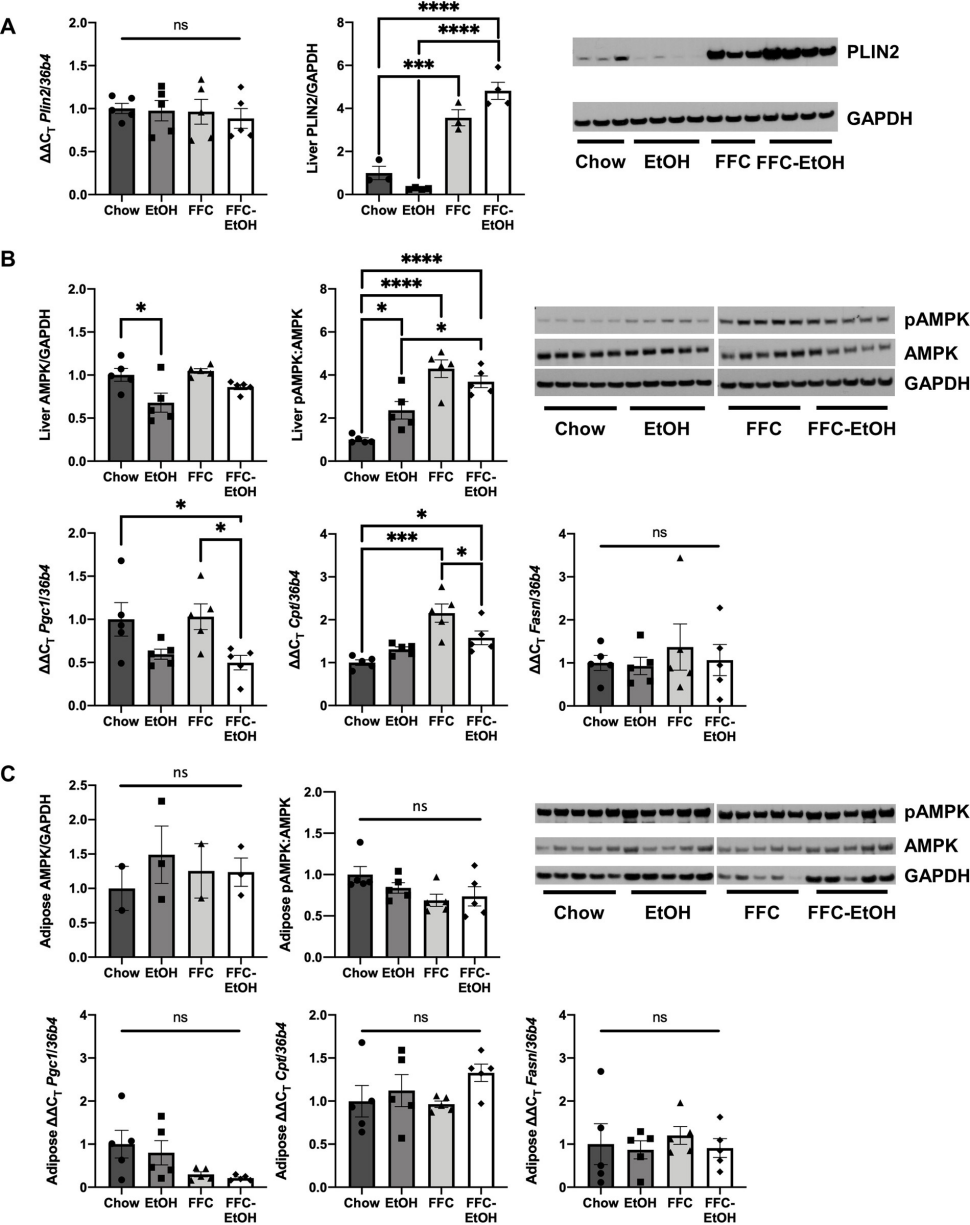
FFC-EtOH increases hepatic, but not WAT, AMPK signaling and fatty acid β-oxidation. (A) Hepatic gene and protein expression for the lipid droplet marker gene Plin2. (B) Hepatic gene and protein expression for AMPK signaling. (C) Gene and protein expression in white adipose tissue for AMPK signaling. Significance was determined by one-way ANOVA followed by Kruskal-Wallis and Dunn’s test (* p ≤ 0.05; ** p ≤ 0.01; *** p ≤ 0.001; **** p ≤ 0.0001; n = 5/group).

Since FFC-EtOH induced significant lipid dysregulation and steatosis compared to Chow diet without a difference in serum TG levels, we hypothesized that these disturbances might be because of effects of the FFC-EtOH diet on lipid droplet biogenesis, fatty acid oxidation, or lipolysis. We examined Perilipin 2 (PLIN2), a lipid droplet protein whose hepatic expression increases in NAFLD and ALD [39] and whose absence we have found to be protective against EtOH-induced hepatic steatosis [18]. Both FFC (P = 0.001) and FFC-EtOH (P < 0.0001) increased hepatic PLIN2 protein expression compared to Chow, but there was no difference in hepatic *Plin2* gene expression between the diet groups (Fig 4A).

AMP-activated protein kinase (AMPK) is a master regulator of fatty acid β-oxidation, whose inhibition by chronic alcohol consumption is associated with increased hepatic lipogenesis [40, 41]. Consistent with several prior studies [42, 43], EtOH decreased total AMPK compared to Chow (Fig 4B). Neither FFC nor FFC-EtOH affected total AMPK levels. FFC increased hepatic AMPK phosphorylation compared to Chow, and there was no difference in pAMPK between FFC-EtOH and FFC in this model of early liver disease. Increased hepatic pAMPK was associated with upregulated carnitine palmitoyltransferase 1 (*Cpt1*) expression, which is involved in fatty acid β-oxidation, in FFC and FFC-EtOH compared to Chow (P = 0.0001 and P = 0.03, respectively). FFC-EtOH downregulated expression of the lipolytic transcription factor PPARγ coactivator 1 alpha (*Pgc1a*), which tended to decrease due to EtOH, and there was no difference in fatty acid synthase (*Fasn*) between the diet groups. These results suggest that FFC-EtOH increases hepatic AMPK signaling and fatty acid β-oxidation, but occur independently of Pgc1a.

**Fig 5:**
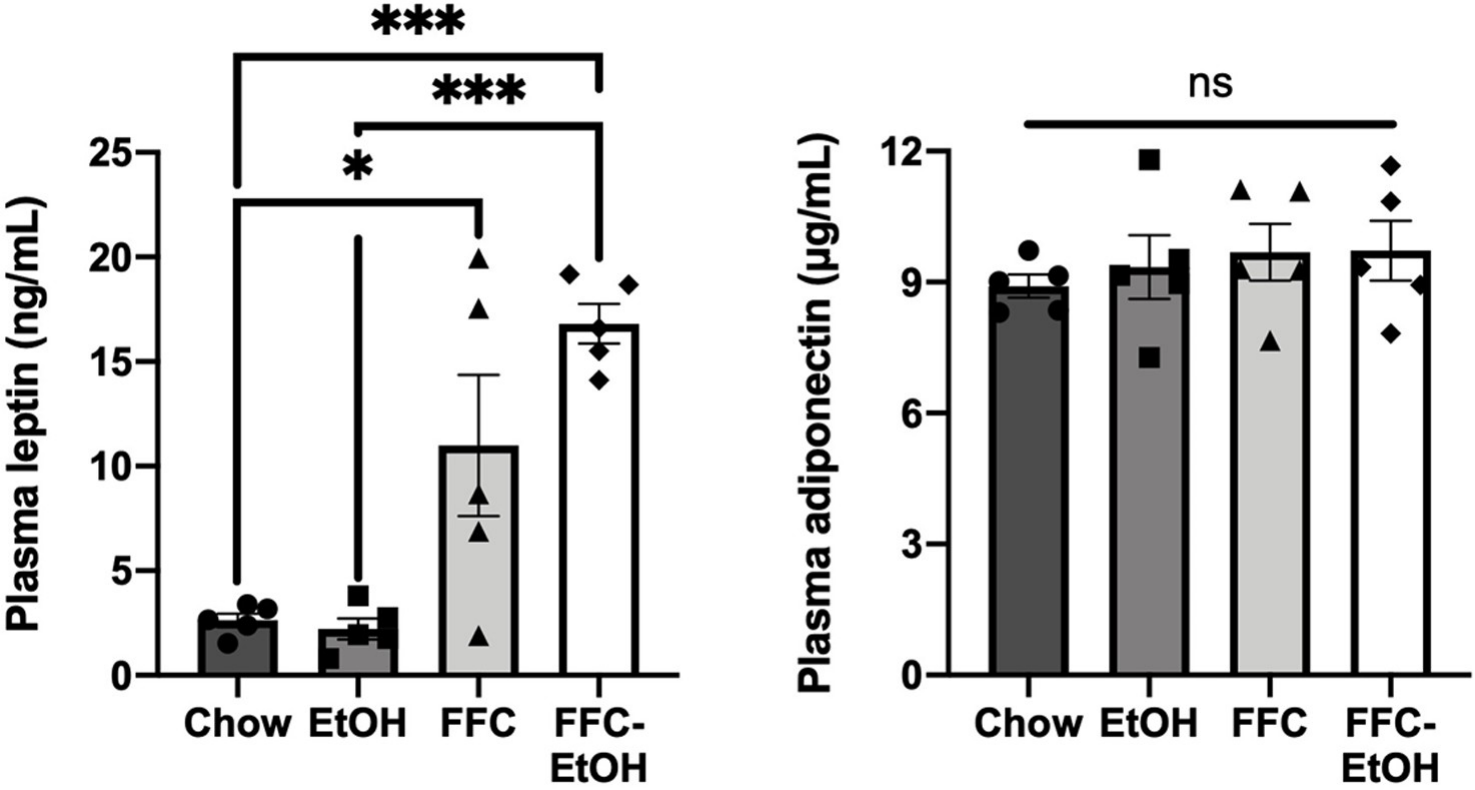
FFC-EtOH increased leptin to a greater extent than FFC. Concentration of the circulating AMPK pathway activators leptin and adiponectin in blood plasma. Significance was determined by one-way ANOVA followed by Kruskal-Wallis and Dunn’s test (* p ≤ 0.05; ** p ≤ 0.01; *** p ≤ 0.001; **** p ≤ 0.0001; n = 5/group).

In WAT, there was no significant difference in total AMPK, AMPK phosphorylation, lipolytic gene expression, or lipogenic gene expression among the diet groups (Fig 4C). Leptin and adiponectin activate the AMPK pathway in both liver and WAT [44], thus the presence of both were analyzed. FFC (P = 0.02) and FFC-EtOH (P = 0.0001) increased plasma leptin levels compared to Chow (Fig 5). There was no significant difference between FFC and FFC-EtOH, but FFC-EtOH tended to increase leptin compared to FFC despite EtOH not doing so compared to Chow. There was no significant difference in adiponectin levels among the diet groups. Thus, there is an association between leptin signaling and FFC-EtOH-induced hepatic lipid dysregulation.

### Alcohol and obesogenic diet synergistically dysregulate insulin signaling and carbohydrate metabolism

**Fig 6:**
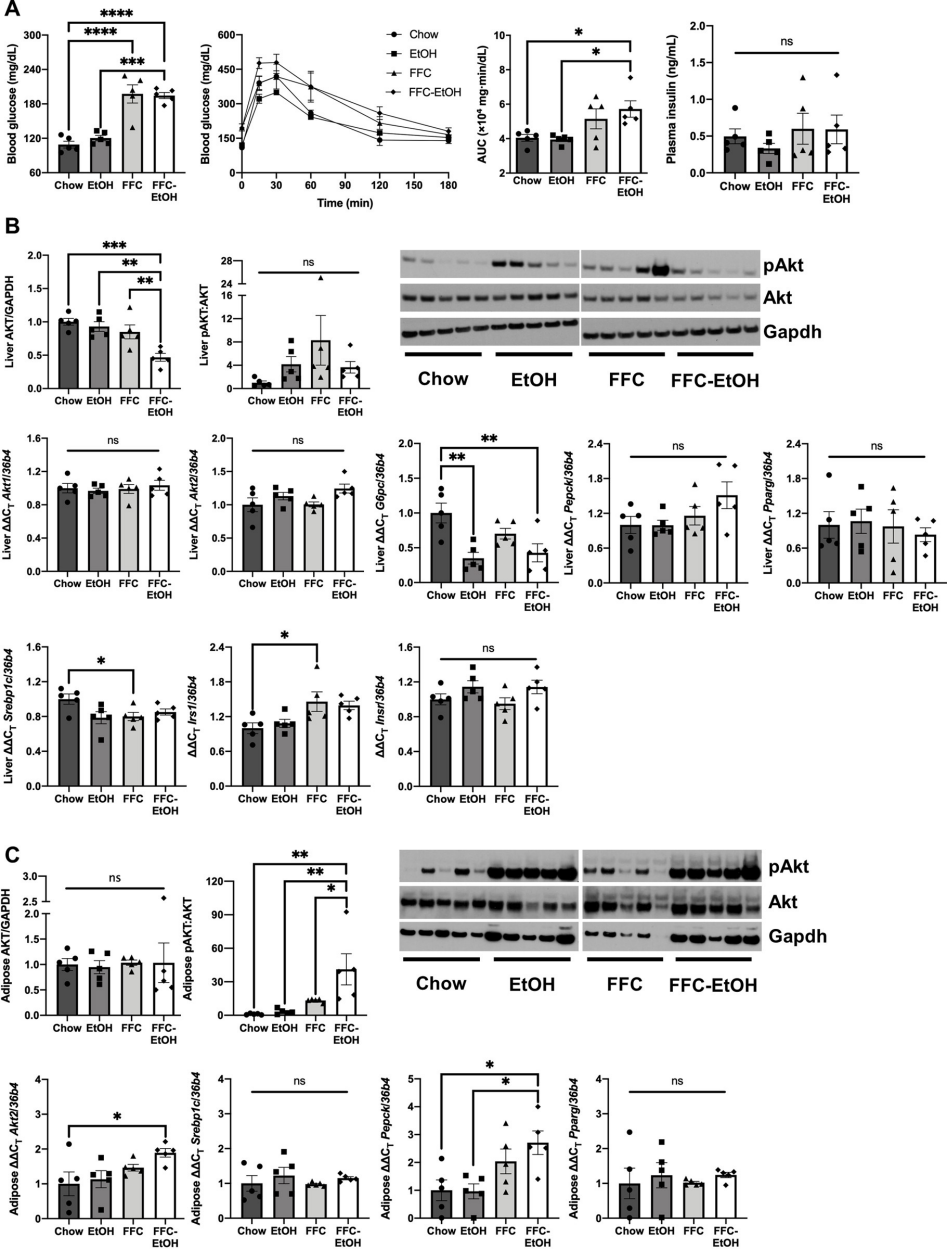
Impairment of glucose clearance due to EtOH, FFC, or FFC-EtOH is primarily from the liver and not by adipose tissue or skeletal muscle. (A) Fasting blood glucose and insulin levels and glucose tolerance test by area under the curve (AUC). (B) Hepatic gene and protein expression markers for glucose uptake and metabolism for the insulin/protein kinase B (AKT) pathway. (C) Gene and protein expression markers in white adipose tissue for glucose uptake and metabolism in the insulin/protein kinase B (AKT) pathway. Significance was determined by one-way ANOVA followed by Kruskal-Wallis and Dunn’s test (* p ≤ 0.05; ** p ≤ 0.01; *** p ≤ 0.001; **** p ≤ 0.0001; n = 5/group).

Insulin resistance is a defining feature of MetS and is associated with increased disease severity in NAFLD and ALD [45]. NAFLD is also a risk factor for type 2 diabetes, in part because hepatic cytokines activate insulin signaling in other tissues [46]. Given the reciprocal relationship between fatty liver disease and insulin sensitivity, we examined glucose clearance and insulin signaling in FFC-EtOH fed mice to determine the role of insulin resistance in SMAFLD pathogenesis. FFC diet increased fasting blood glucose levels compared to Chow (197 ± 16 mg/dL and 109 ± 6 mg/dL, respectively), but there was no difference between FFC and FFC-EtOH (195 ± 5 mg/dL) (Fig 6A). However, FFC-EtOH induced glucose intolerance compared to Chow (P = 0.04), as evidenced by an increase in GTT area under the curve (AUC) values. Neither FFC alone nor EtOH alone significantly changed the AUC compared to Chow. As there was no significant difference in fasting insulin levels between the dietary groups, these observations demonstrate that FFC-EtOH impairs glucose utilization compared with either FFC or EtOH alone.

Since FFC-EtOH significantly impaired glucose clearance, we hypothesized that FFC-EtOH treatment dysregulates the insulin/protein kinase B (AKT) pathway, which controls glucose uptake and metabolism. FFC-EtOH decreased total hepatic AKT protein levels compared to Chow, EtOH, and FFC (Fig 6B; P = 0.0007, P = 0.003, and P = 0.01, respectively) and tended to reduce pAKT compared to FFC. The differences in AKT in the FFC-EtOH group were associated with a tendency for increased phosphoenolpyruvate carboxykinase (*Pepck*) compared to Chow. FFC-EtOH did not significantly affect the gene expression of sterol regulatory element-binding protein (*Srebp1c*) or peroxisome proliferator-activated receptor gamma (*Pparg*), both of which are lipogenic transcription factors that are implicated in insulin resistance, compared to Chow. FFC upregulated hepatic insulin receptor substrate 1 (*Irs1*) expression compared to Chow, and there was no difference in insulin receptor (*Insr*) between the diet groups.

The expression of genes in gluconeogenesis were quantified by RNA-seq (S1 Fig). Among 71 genes in the gluconeogenic pathway, 29 were significantly regulated by EtOH, FFC, or FFC-EtOH. Glucose-6-phosphate, catalytic (G6pc) was increased in EtOH only, indicating that there is active glucose production. Opposing active gluconeogenesis, glucose phosphorylating enzymes hexokinase 1 (Hk1) and Hk2 expression was increased by FFC-EtOH and Hk3 was increased by both FFC and FFC-EtOH. Expression of pyruvate kinase liver and red blood cell (Pklr), which produces pyruvate, and pyruvate carboxylase (Pcx), which converts pyruvate to oxaloacetate, was increased or tended to increase by FFC and FFC-EtOH.

In WAT, FFC-EtOH increased pAKT compared to Chow, EtOH, and FFC (Fig 6C; P = 0.003, P = 0.006, and P = 0.05, respectively). There was no difference in *Srebp1c*, *Pparg*, *Irs1*, or *Insr* between the diet groups. Finally, in skeletal muscle, there was no difference in AKT, pAKT, glucogenic genes, or lipogenic genes among the diet groups (S2 Fig). These results suggest that the observed impairment in glucose clearance is not driven by dysregulation of muscular or WAT insulin signaling, which contrasts with the known emergence of insulin resistance in skeletal muscle [47, 48] and adipose tissue [49] in NAFLD.

**Fig 7:**
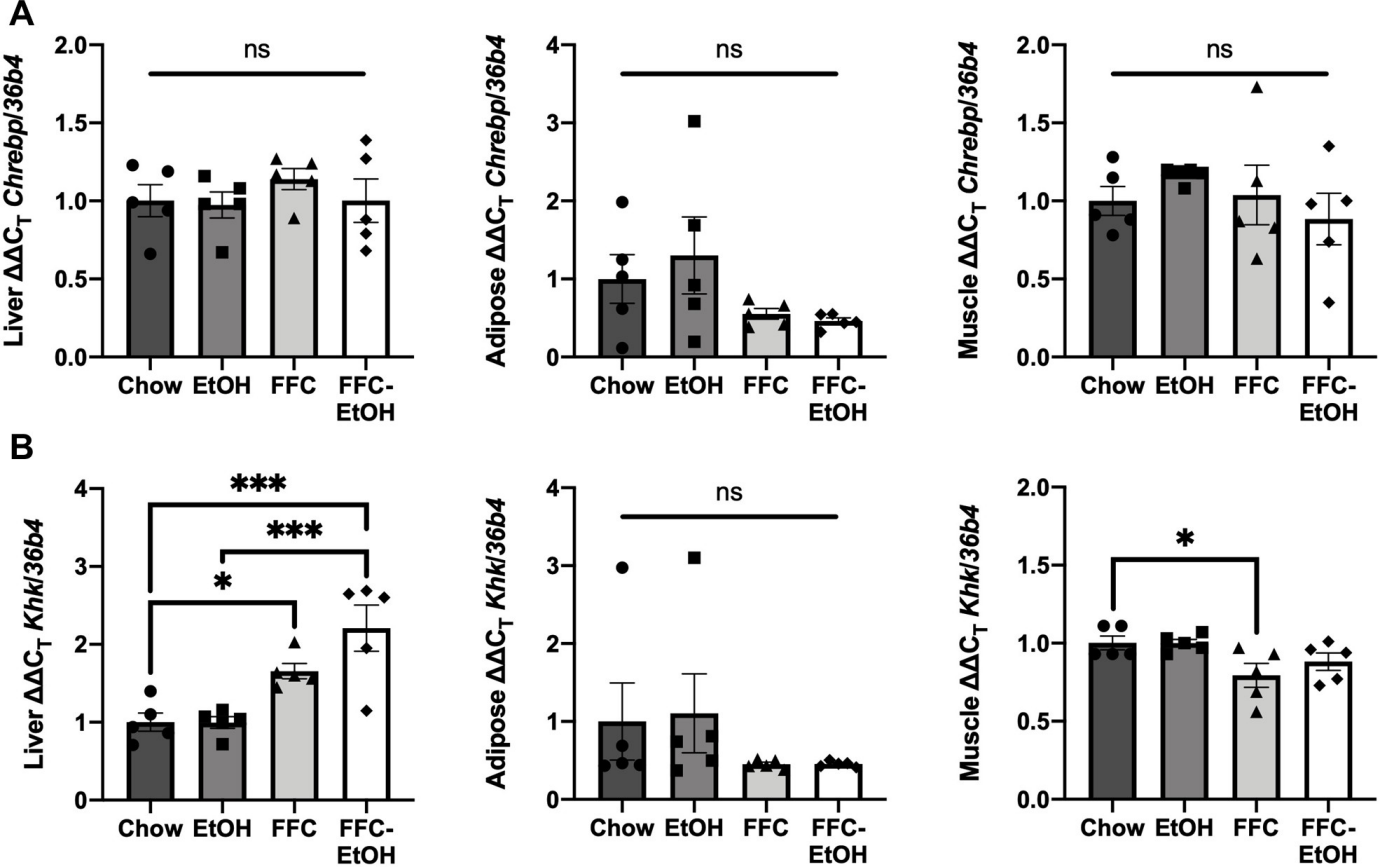
Concomitant FFC-EtOH increases hepatic fructose metabolism. The mRNA gene expression for fructose metabolism markers in liver, white adipose tissue, and skeletal muscle for (A) the carbohydrate-responsive element-binding protein (CHREBP) and (B) ketohexokinase (KHK). Significance was determined by one-way ANOVA followed by Kruskal-Wallis and Dunn’s test (* p ≤ 0.05; ** p ≤ 0.01; *** p ≤ 0.001; **** p ≤ 0.0001; n = 5/group).

Fructose metabolism was also examined because fructolysis and insulin signaling indirectly, but reciprocally regulate each other. Fructose enters hepatocytes via the insulin-independent transporter GLUT2 and is then phosphorylated by ketohexokinase (KHK). Fructose-1-phosphate is ultimately converted into glyceraldehyde-3-phosphate that is directed to the glycolysis pathway. *Glut2*, *Khk*, and *G6pc* are regulated by carbohydrate-responsive element-binding protein (CHREBP) [50, 51]. CHREBP is upregulated in hyperinsulinemia, while CHREBP knockout is associated with insulin resistance [52]. Therefore, we hypothesized that the impaired glucose tolerance by FFC-EtOH would be associated with fructose metabolism dysregulation. However, there was no significant difference in *Chrebp* expression between the diet groups in liver, WAT, or muscle (Fig 7A). Interestingly, FFC upregulated hepatic *Khk* and downregulated muscular *Khk* expression compared to Chow, but there was no difference in WAT *Khk* between the diet groups (Fig 7B). FFC-EtOH tended to increase hepatic *Khk* compared to FFC, but the difference was not significantly different. These results suggest that combined FFC-EtOH possibly contributes to insulin resistance by increasing hepatic fructose metabolism, a known contributor to hepatic *de novo* lipogenesis [53].

### Alcohol and obesogenic diet enriched mRNA gene expression of immune and metabolic responses

**Fig 8:**
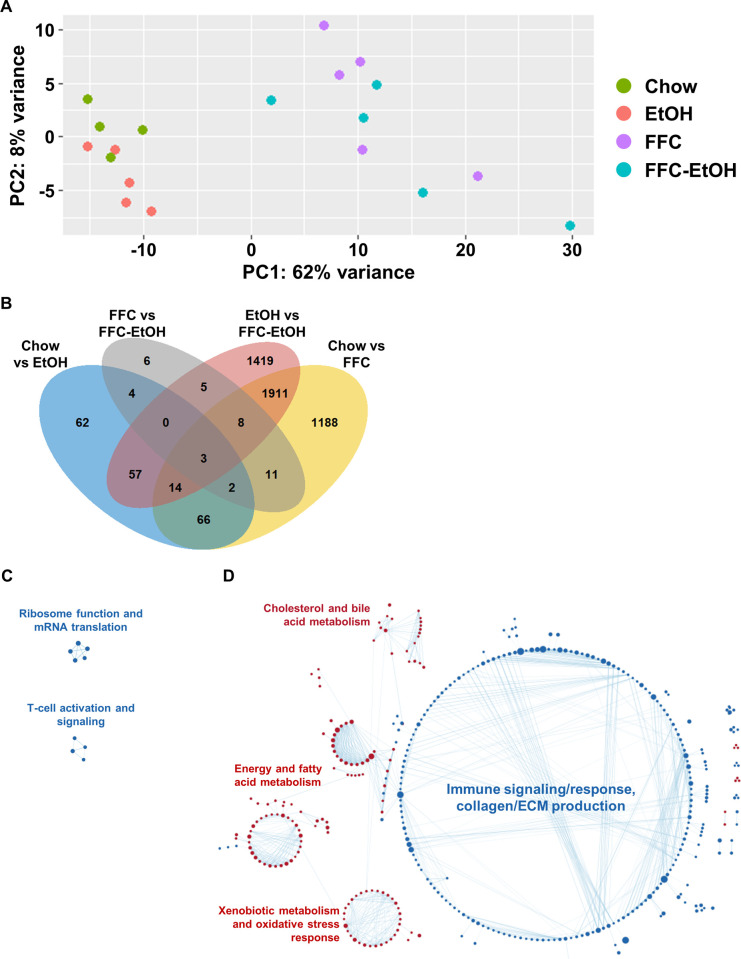
Global RNA-seq analysis indicates more genes and pathways are altered due to FFC compared to EtOH. (A) Principal component analysis of the hepatic genes expressed in at least one sample. A gene was considered expressed if the TPM in one sample was greater than or equal to 10. (B) A four-way Venn diagram describing the number of differentially expressed protein-coding genes between the different diet comparison groups. A protein-coding gene was considered expressed if the average expression (TPM) in one group was greater than or equal to 10. Differential expression was considered at the FDR-BH < 0.05 (Benjamini-Hochberg false discovery rate). Gene Set Enrichment Analysis (GSEA) visualization from Cytoscape for (C) FFC and (D) EtOH groups compared to the FFC-EtOH FFC group. A gene set pathway is considered enriched for FDR < 0.05. Red nodes are enriched in FFC or EtOH groups, whereas blue is enriched in the FFC-EtOH group. (n = 3-5/group).

To further characterize and define the synergistic effects of FFC-EtOH in the liver as well as pathways affected by EtOH or FFC, we examined hepatic transcription by RNA-sequencing. Among the 21,727 protein-coding genes, 17,249 were considered expressed with a TPM ≥ 10 in at least one sample. Principle component analysis (PCA) of the normalized transcript abundance of the expressed genes identified two distinct groups based on chow type: 1) Chow and EtOH and 2) FFC and FFC-EtOH (Fig 8A). Among the expressed genes, 4,756 were differentially regulated (FDR-BH < 0.05) by one of four comparisons: 1) Chow versus EtOH, 2) Chow versus FFC, 3) EtOH versus FFC-EtOH, and 4) FFC versus FFC-EtOH (Fig 8B). To examine the synergistic effects of FFC and EtOH, the Venn diagram shows 16 genes differentially regulated by only the co-exposure of FFC-EtOH; EtOH or FFC alone are insufficient to alter the expression of these 16 genes. Compared to concomitant exposure, EtOH differentially regulated 39 genes whereas FFC differentially regulated 3,417 genes. This is also demonstrated in the 1-way hierarchical clustered heatmap with most genes regulated by exposure to FFC and only a subset of genes regulated by EtOH (S3 Fig). Relative to EtOH, FFC has greater impact on hepatic gene expression.

Pathway analysis by Gene Set Enrichment Analysis (GSEA) and visualization by Cytoscape was used with the normalized expression of the protein coding genes to examine the synergistic effects of EtOH and FFC. Comparing the effect of FFC relative to the concomitant exposure of EtOH-FFC (FFC versus FFC-EtOH), GSEA categorized gene expression differences into 17 enriched pathways (FDR < 0.05; S1 Table) focused on two major functions: 1) ribosome function and 2) T-cell activation and signaling (Fig 8B). This indicates that EtOH can be attributed to the increased expression of genes involved in mRNA translation and T-cell activation in the concomitant exposure of EtOH and FFC. Comparing the effect of EtOH relative to the concomitant exposure of EtOH-FFC (EtOH versus FFC-EtOH), GSEA identified 483 enriched pathways (S1 Table). The FFC exposure can be attributed to the decreased expression of genes related to cholesterol and bile acid metabolism, energy and fatty acid metabolism, and xenobiotic metabolism and oxidative stress response whereas genes with increased expression were enriched in pathways related to immune signaling/response and collagen or extracellular matrix (ECM) production (Fig 8D). These results from the hepatic transcriptome suggest that the synergistic effects of FFC and EtOH alter lipid and cholesterol metabolism while creating an enriched expression of an inflammatory environment.

## Discussion

In this study, mice were fed an FFC diet and administered chronic-binge EtOH to determine the physiological effects of concomitant obesogenic diet and alcohol consumption in early-stage SMAFLD. Combined FFC-EtOH treatment induced steatosis, lipid dysregulation, and glucose intolerance compared to FFC or EtOH alone. Furthermore, although FFC-EtOH did not induce histologic steatohepatitis or fibrosis, FFC-EtOH increased pro-fibrotic gene expression. FFC-EtOH increased body weight, promoted hepatomegaly, and increased ALT activity compared to Chow. FFC-EtOH also tended to promote more severe steatosis compared to FFC, despite the lack of steatosis in EtOH-fed mice. While combined obesogenic diet and EtOH consumption may have an additive, synergistic, or antagonistic relationship with regards to SMAFLD severity, the results from this study suggest a possible synergistic relationship between FFC and EtOH.

In our study, FFC-EtOH and FFC diets were also associated with expression of antioxidant defense and immune response genes. Oxidative stress is certainly part of SMAFLD pathophysiology, as concomitant obesity and EtOH in rats impairs antioxidant capacity [54]. Previous studies in mice suggest that concomitant HFD and EtOH induces pro-inflammatory gene expression [35] and M1 phenotype in Kupffer cell [55]. However, other studies suggest that EtOH alleviates diet-induced pro-inflammatory signaling [23, 56]. Thus, while the role of alcohol consumption on oxidative stress and immune response in MetS and NAFLD is not fully understood, the present study suggests that concomitant obesity and EtOH dysregulates the cellular stress response more than EtOH alone in the early stages of SMAFLD.

FFC-EtOH promoted lipid dysregulation as evidenced by higher hepatic TG levels compared to EtOH; increased hepatic and plasma long-chain ceramides; and upregulated the lipid droplet protein, PLIN2, consistent with our prior observation that ceramides positively regulate PLIN2 and influence lipid droplet accumulation [57, 58]. FFC-EtOH also induced hepatic AMPK activation and upregulated the oxidative gene Cpt1, perhaps as a compensatory mechanism to combat steatosis.

FFC-EtOH impairs glucose tolerance without concomitant hyperinsulinemia more than either dietary component alone, a consequence of both dysregulated hepatic insulin signaling and carbohydrate metabolism. The lack of hyperinsulinemia (a marker of pancreatic beta cell function) or impaired WAT insulin signaling suggests that the hepatic impairment of insulin signaling precedes extra-hepatic manifestations of glucose dysregulation in early SMAFLD. That our results differ from that of Alwahsh et al. who observed hyperinsulinemia in rats who develop hepatic inflammation and fibrosis upon being chronically fed a Lieber-DeCarli ethanol and fructose diet [59] supports our conclusion that early SMAFLD is physiologically distinct from more advanced stages of liver disease.

Finally, examination of the hepatic transcriptome in early SMAFLD induced by an FFC-EtOH diet demonstrates the engagement of several additional pathways. Namely, the addition of FFC to an EtOH diet inhibited energy, lipid, bile acid, and xenobiotic metabolic pathways. This effect on xenobiotic metabolism is especially concerning as ethanol requires such pathways for its metabolism to less toxic metabolites. The FFC diet combined with EtOH also augments immune and extracellular matrix genes, the activation of which may reflect the earliest of changes in the progression of early stage SMAFLD to steatohepatitis and fibrosis.

In summary, we report our findings of an experimental mouse model of early stage SMAFLD using a chronic FFC and ethanol diet with weekly binge ethanol ingestion, mimicking the most deleterious pattern of alcohol intake for liver health. This model recapitulates histologic steatosis without histologic inflammation or fibrosis and identifies several metabolic, inflammatory, and fibrotic pathways that are already engaged at this early stage of disease. These early signals are opportunities for the identification of metabolic targets that may be leveraged for prevention of disease progression and ultimately for mitigation of advanced liver injury in SMAFLD.

## Supporting information

S1 Fig Gluconeogenesis individual gene expression. Hepatic RNA expression for genes involved in gluconeogenesis and significantly regulated by EtOH, FFC, or FFC-EtOH. Significance was determined by using DESeq2 with FDR/Benjamini-Hochberg with EtOH and FFC compared to Chow and FFC-EtOH compared to EtOH (FDR-BH; * p ≤ 0.05; n = 5/group). No genes had differential expression between FFC-EtOH and FFC.
(TIF)

S2 Fig Skeletal muscle glucose and metabolism markers. Gene and protein expression markers in skeletal muscle for glucose uptake and metabolism in the insulin/protein kinase B (AKT) pathway. Significance was determined by one-way ANOVA followed by Kruskal-Wallis and Dunn’s test (* p ≤ 0.05; ** p ≤ 0.01; *** p ≤ 0.001; **** p ≤ 0.0001; n = 5/group).
(TIF)

S3 Fig Heatmap of expressed hepatic genes. A one-way hierarchical clustering dendrogram showing the relative expression patterns of expressed hepatic genes that are significant and expressed in at least one diet group (Chow, EtOH, FFC, or FFC-EtOH). Data were normalized and are expressed as z scores (n = 3-5/group).
(TIF)

S1 Table Antibodies for western blot, primer sequences, RNAseq transcript counts, pathway enrichment for FFC compared to FFC-EtOH, and pathway enrichment for EtOH compared to FFC-EtOH. This file contains four sets of tables as follows: 1) List of target proteins, antibodies, and antibody source that were used for Western Blot analysis. 2) List of qPCR primer sequences. 3) RNAseq transcript counts data used for the present study. 4) Gene Set Enrichment Analysis (GSEA) results from FFC compared to FFC-EtOH. A gene set pathway was considered enriched for FDR < 0.05. A positive enrichment score (ES) or nominal ES (NES) is enriched in the FFC group, whereas a negative ES or NES is enriched in the FFC-EtOH group. 5) Gene Set Enrichment Analysis (GSEA) results from EtOH compared to FFC-EtOH. A gene set pathway was considered enriched for FDR < 0.05. A positive enrichment score (ES) or nominal ES (NES) is enriched in the EtOH group, whereas a negative ES or NES is enriched in the FFC-EtOH group.
(XLSX)

S1 Raw images (PDF)
