# Supplementary figures and images for: Concomitant western diet and chronic-binge alcohol dysregulate hepatic metabolism
Source: PLoS One. 2023 May 3;18(5):e0281954. doi: 10.1371/journal.pone.0281954 (PMC10155975; doi:10.1371/journal.pone.0281954)

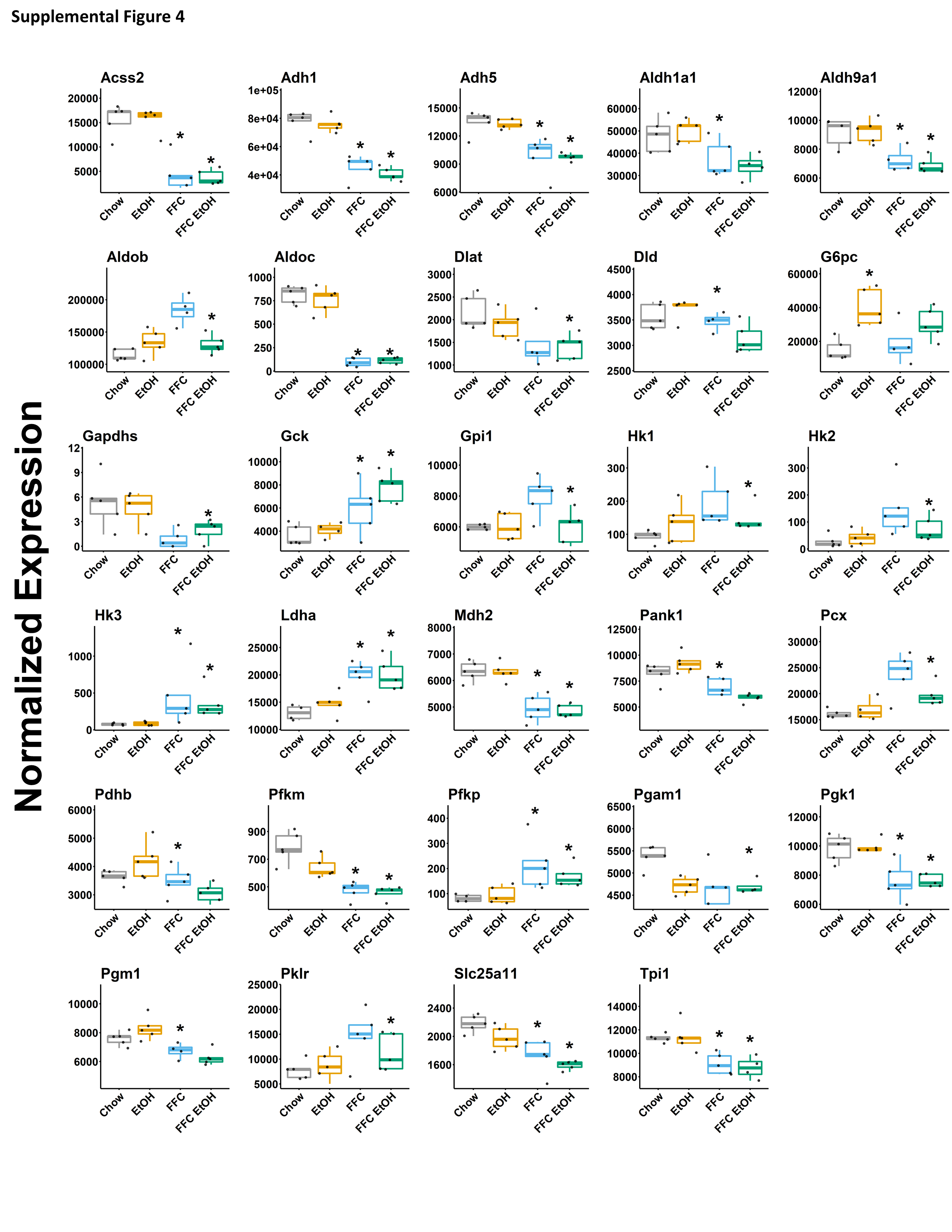

Supplement: S1 Fig — Hepatic RNA expression for genes involved in gluconeogenesis and significantly regulated by EtOH, FFC, or FFC-EtOH. Significance was determined by using DESeq2 with FDR/Benjamini-Hochberg with EtOH and FFC compared to Chow and FFC-EtOH compared to EtOH (FDR-BH; * p ≤ 0.05; n = 5/group). No genes had differential expression between FFC-EtOH and FFC. (TIF) [file pone.0281954.s001.tif]

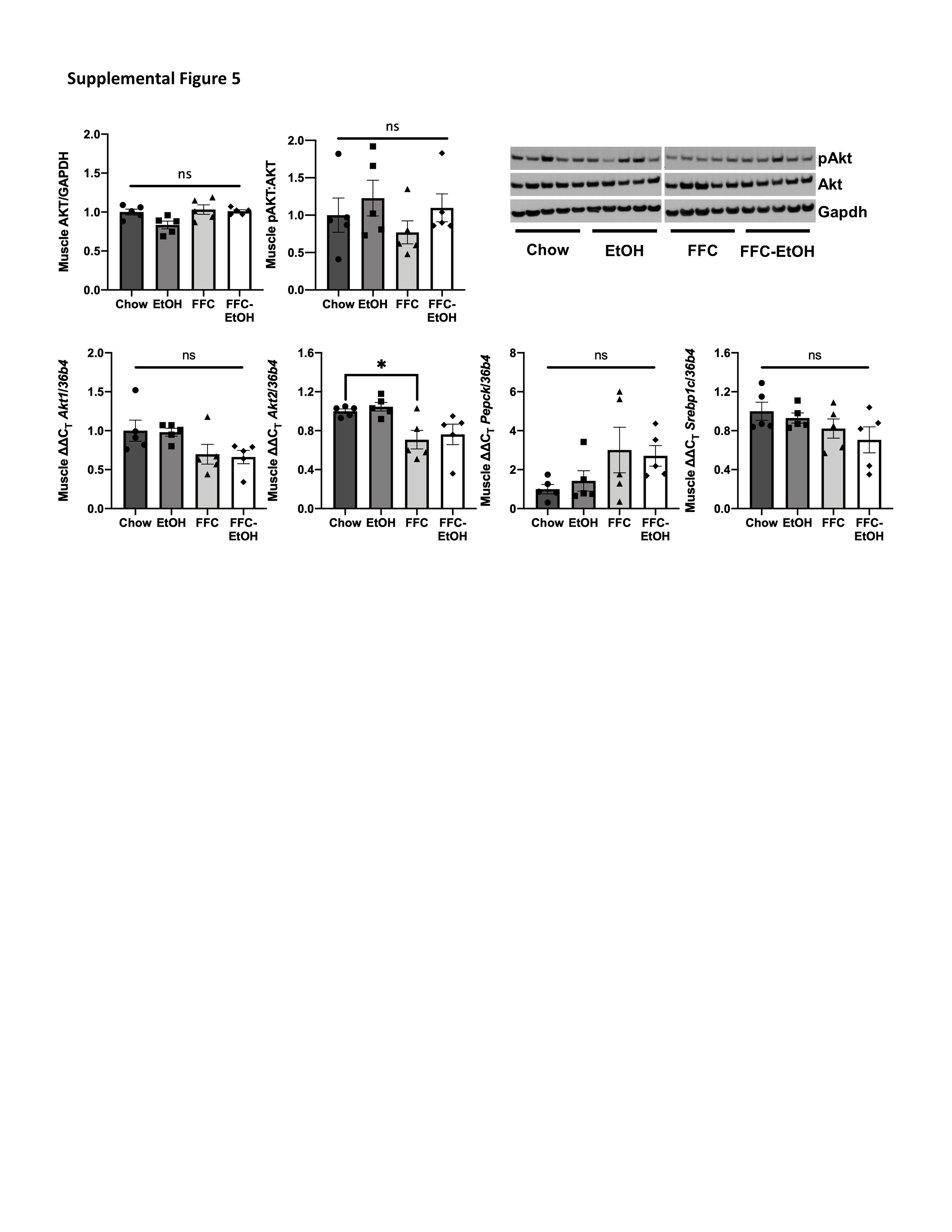

Supplement: S2 Fig — Gene and protein expression markers in skeletal muscle for glucose uptake and metabolism in the insulin/protein kinase B (AKT) pathway. Significance was determined by one-way ANOVA followed by Kruskal-Wallis and Dunn’s test (* p ≤ 0.05; ** p ≤ 0.01; *** p ≤ 0.001; **** p ≤ 0.0001; n = 5/group). (TIF) [file pone.0281954.s002.tif]

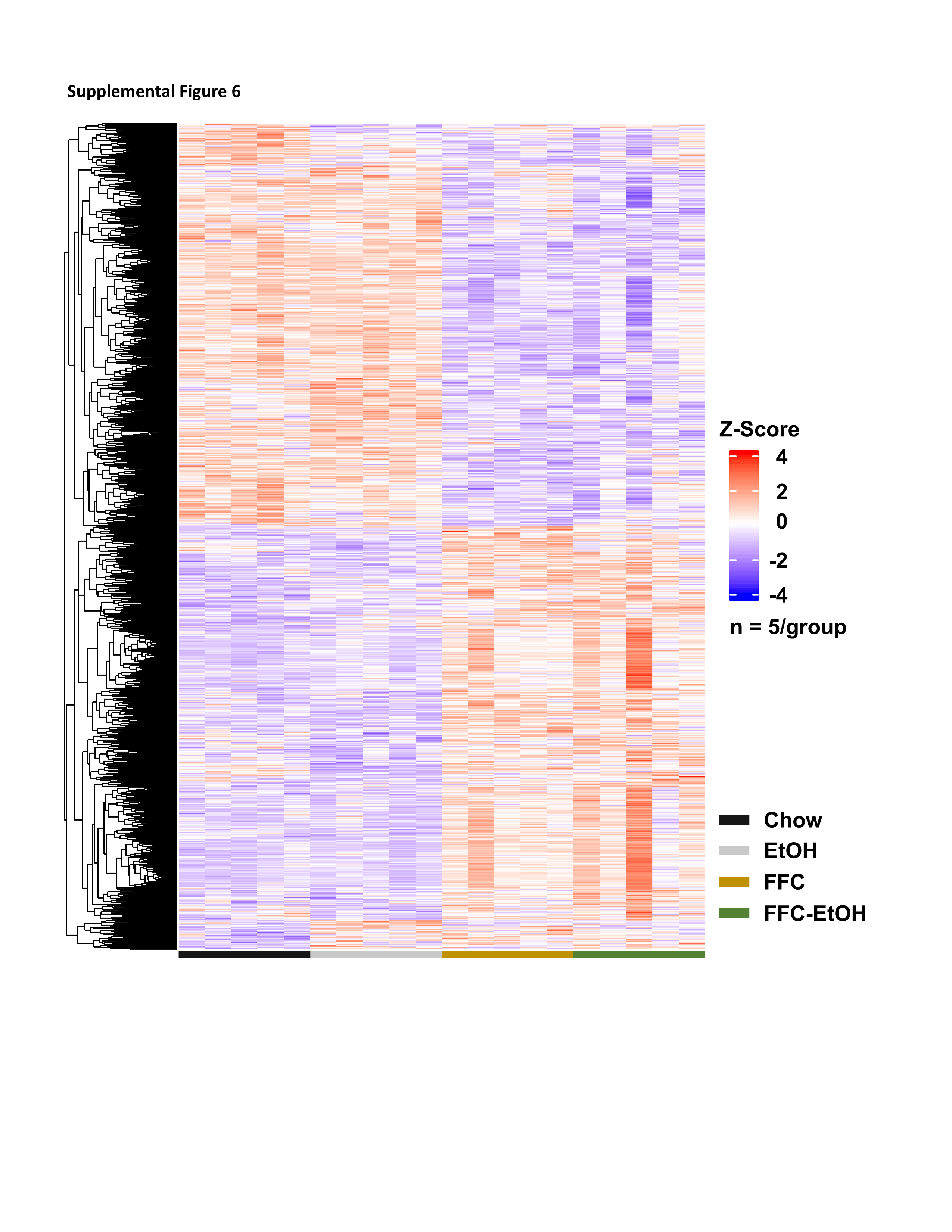

Supplement: S3 Fig — A one-way hierarchical clustering dendrogram showing the relative expression patterns of expressed hepatic genes that are significant and expressed in at least one diet group (Chow, EtOH, FFC, or FFC-EtOH). Data were normalized and are expressed as z scores (n = 3-5/group). (TIF) [file pone.0281954.s003.tif]
