# Supplementary material for: Concomitant western diet and chronic-binge alcohol dysregulate hepatic metabolism
Source: PLoS One. 2023 May 3;18(5):e0281954. doi: 10.1371/journal.pone.0281954 (PMC10155975; doi:10.1371/journal.pone.0281954)

Figure 4A

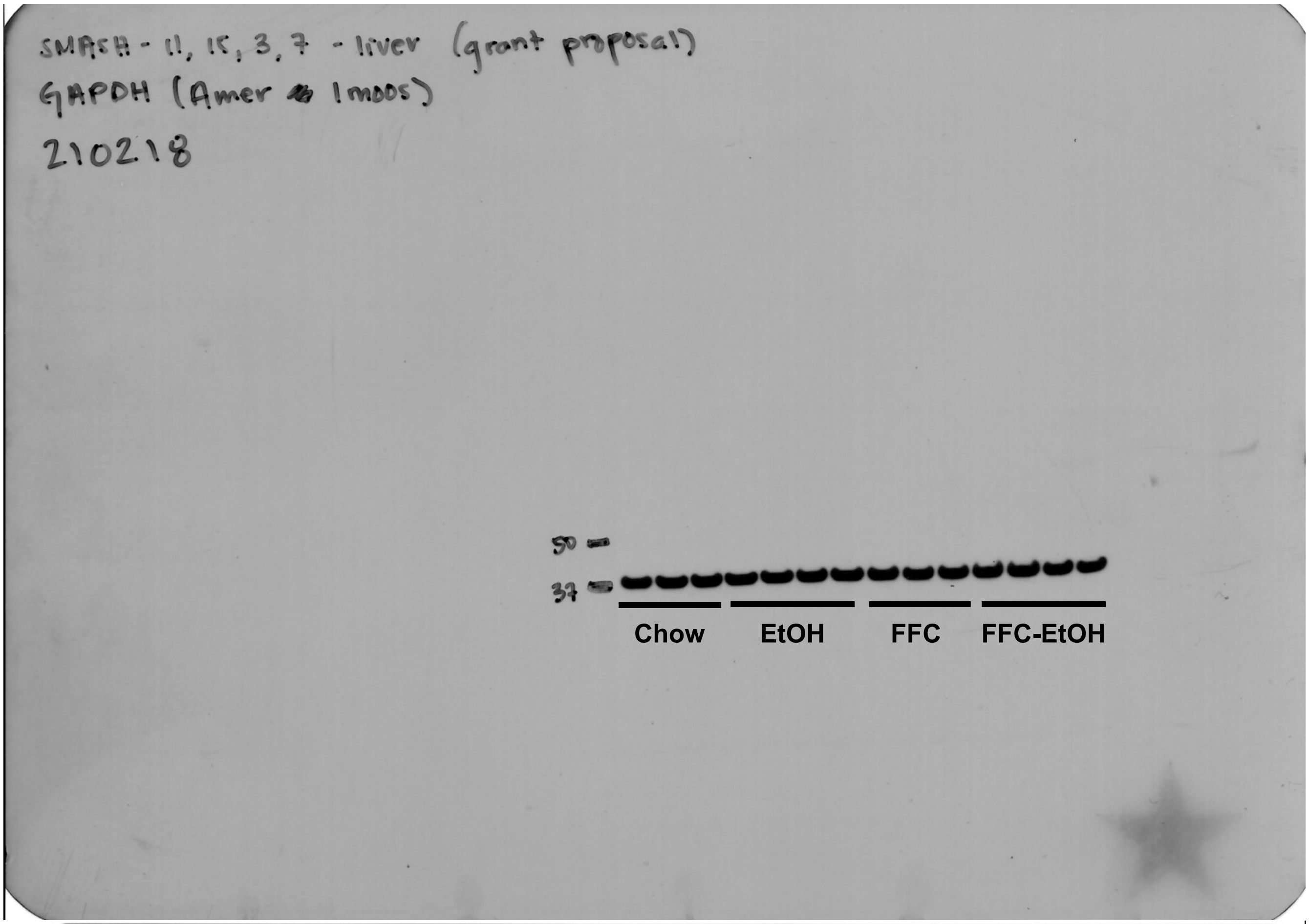
SMASH - 11, 15, 3, 7 - liver (grant proposal)
GAPDH (Amer 1m00s)
210218
50
37
Chow
EtOH
FFC
FFC-EtOH

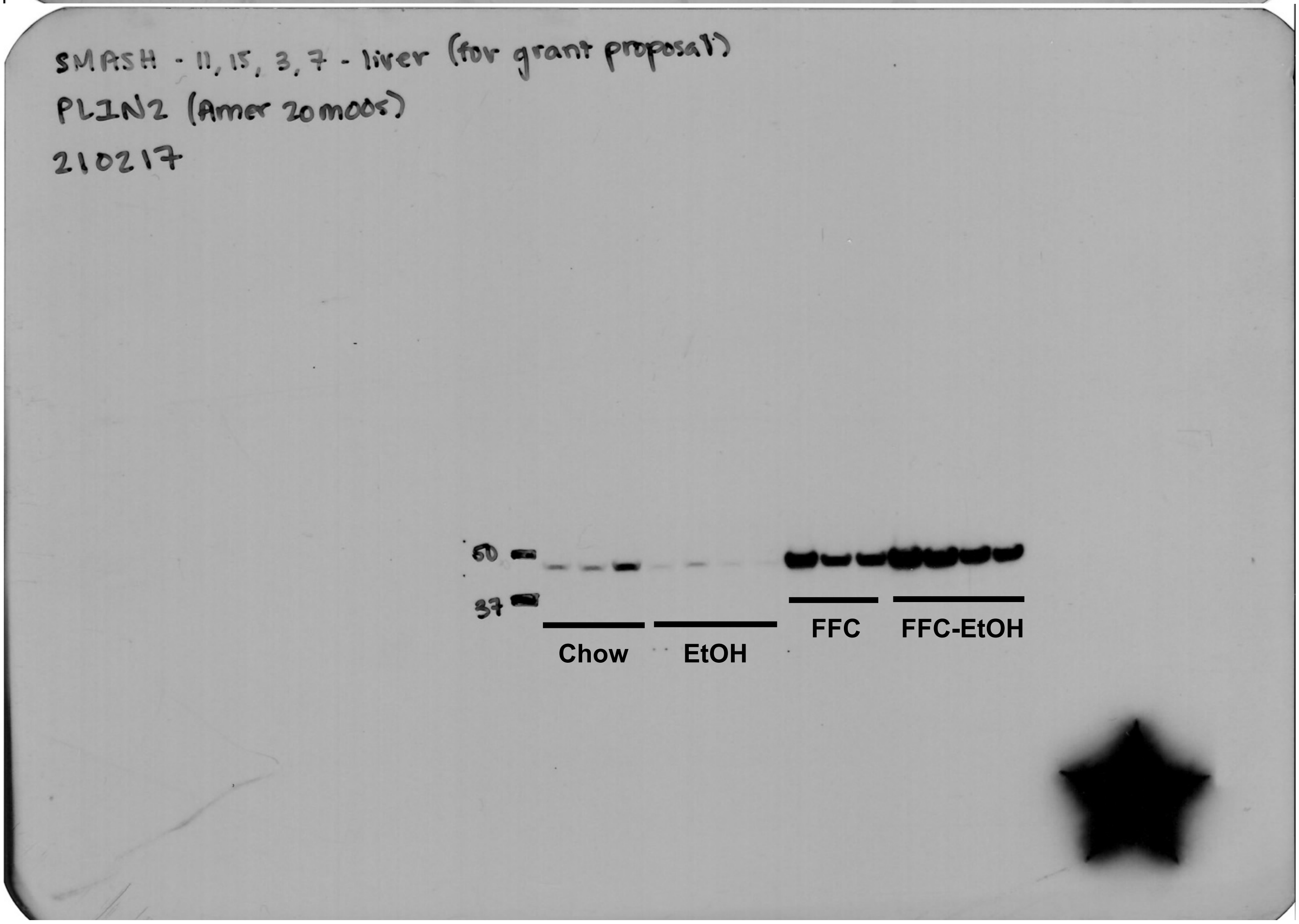
SMASH - 11, 15, 3, 7 - liver (for grant proposal)
PLIN2 (Amer 20m00s)
210217
50
37
Chow
EtOH
FFC
FFC-EtOH

Figure 4B

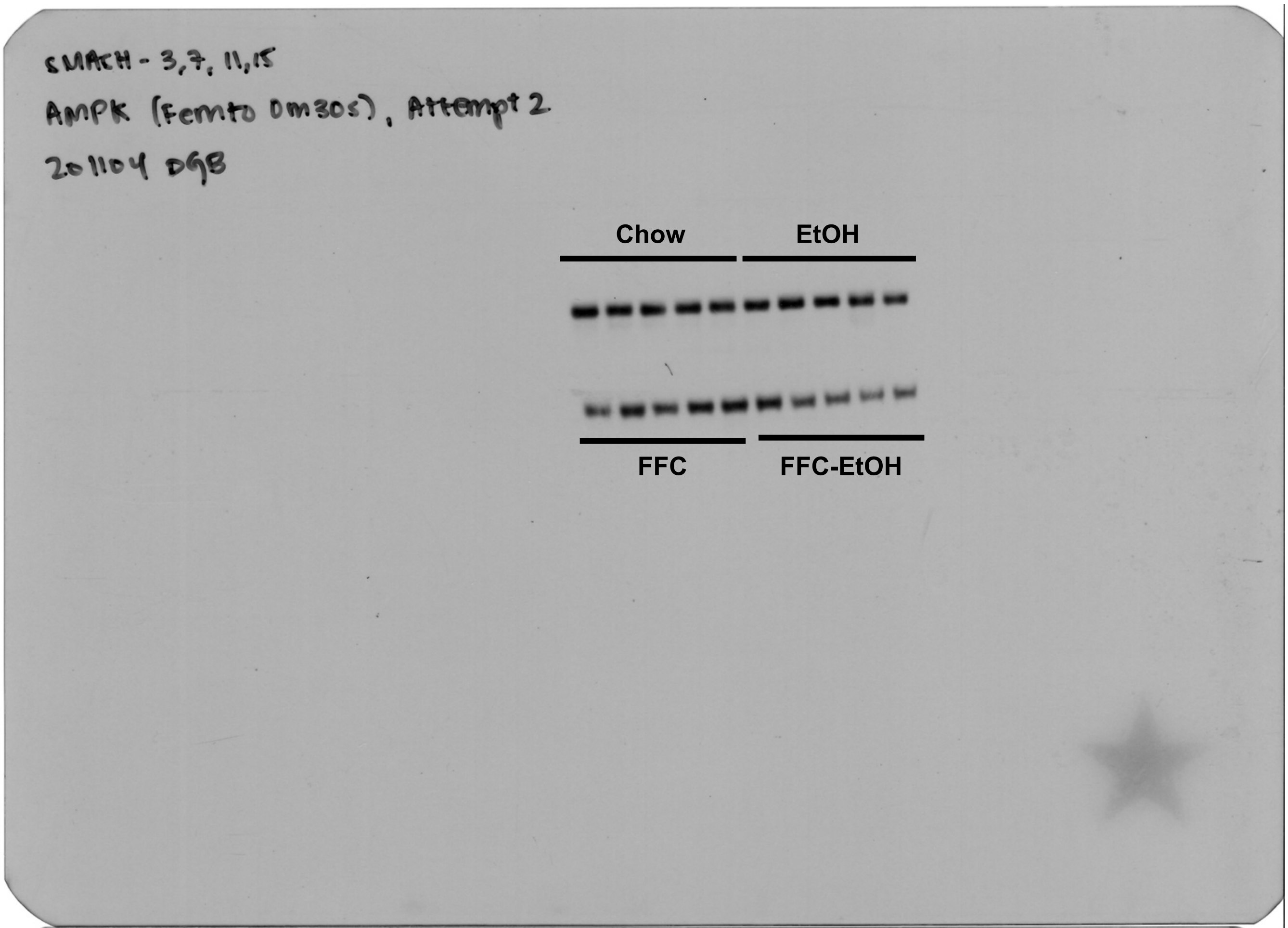
SMASH - 3, 7, 11, 15
AMPK (Femto 0m30s), Attempt 2
201104 DGB
Chow
EtOH
FFC
FFC-EtOH

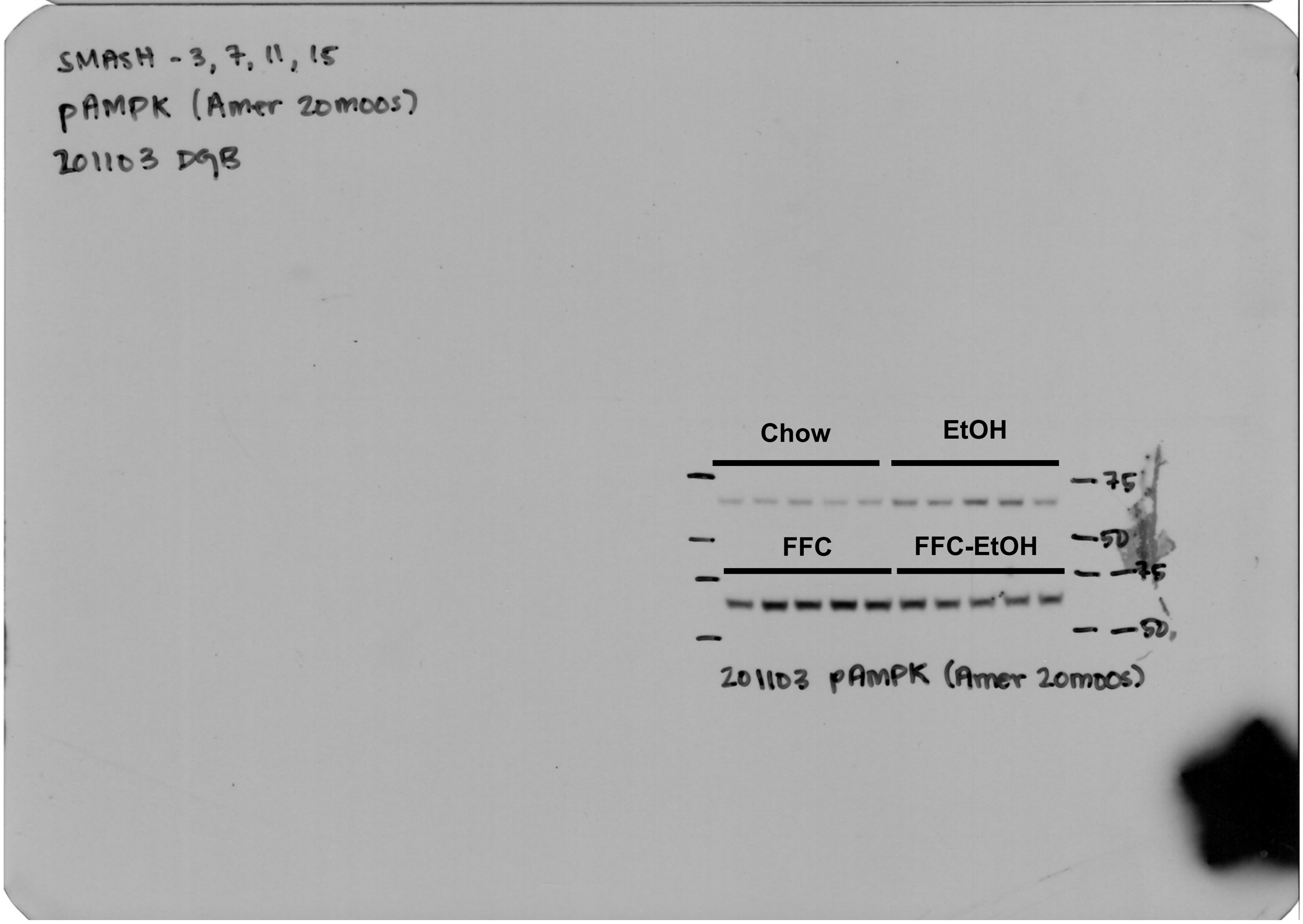
SMASH - 3, 7, 11, 15
pAMPK (Amer 20m00s)
201103 DGB
Chow
EtOH
75
FFC
FFC-EtOH
50
75
50
201103 pAMPK (Amer 20m00s)

Figure 4B

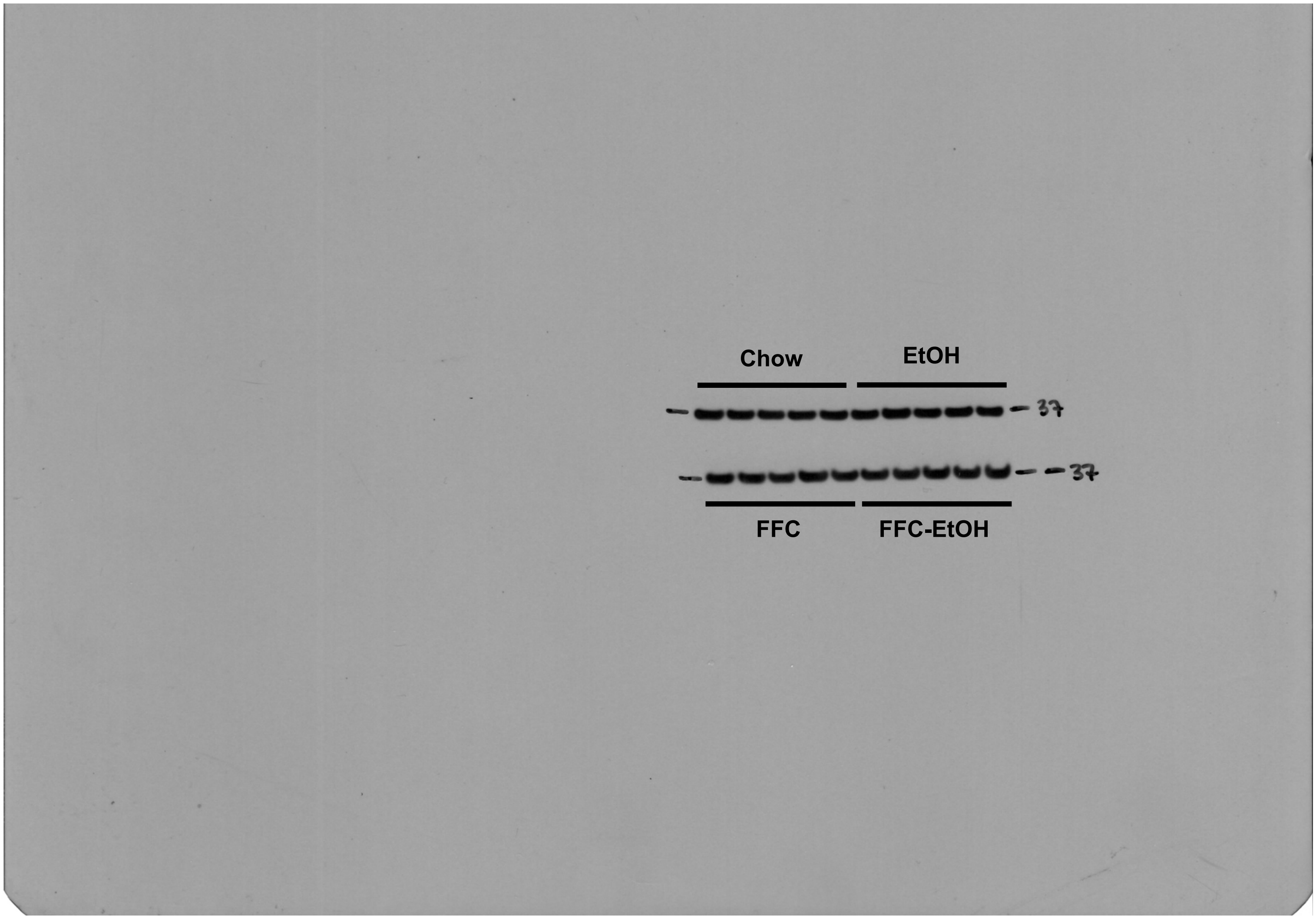

Figure 4C

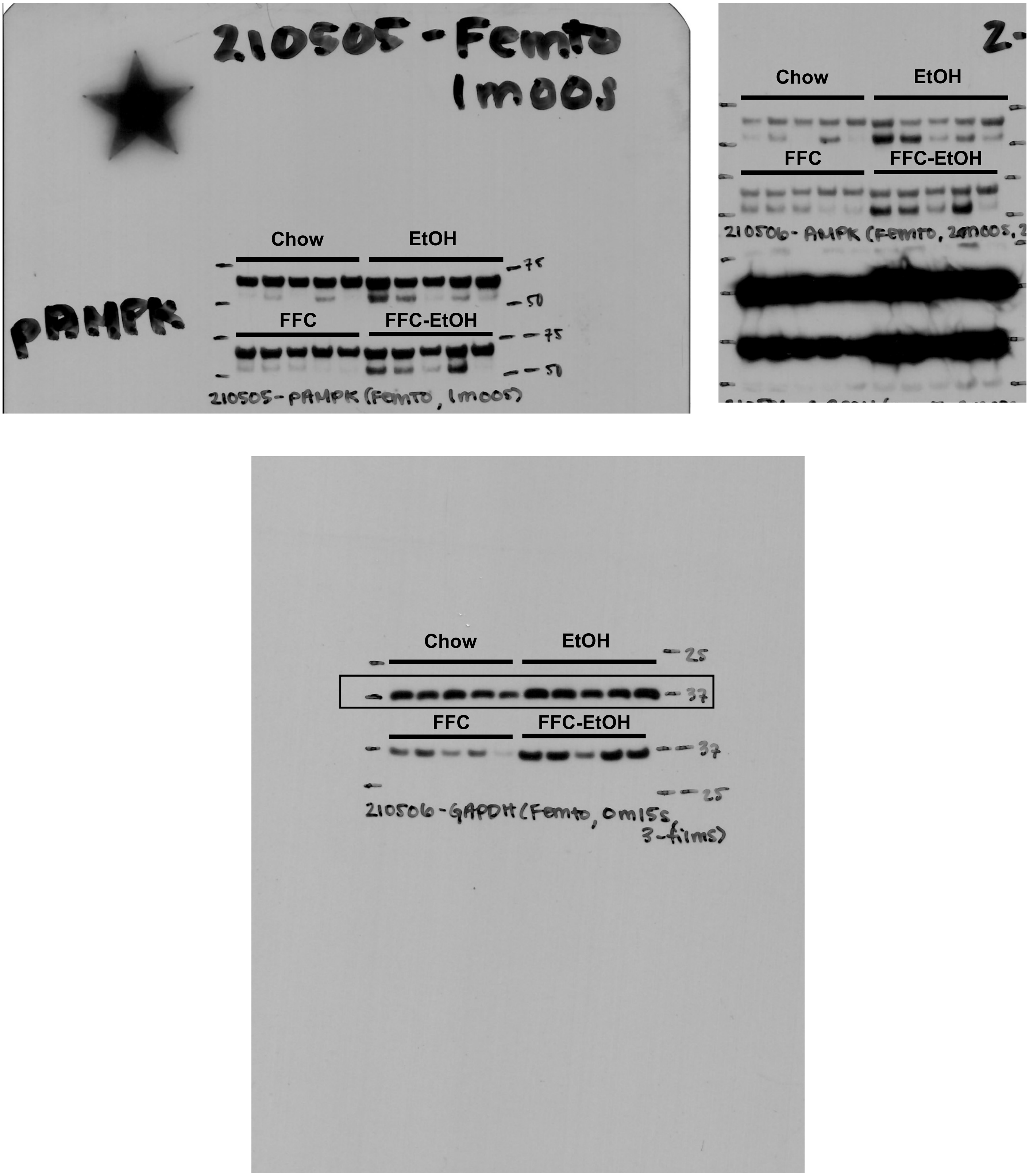
210505 - Femto
1m00s
pAMPK
Chow
EtOH
FFC
FFC-EtOH
75
50
210505 - pAMPK (Femto, 1m00s)
210506 - AMPK (Femto,
210506 - GAPDH (Femto, 0m15s, 3-films)
25
37

Figure 6B

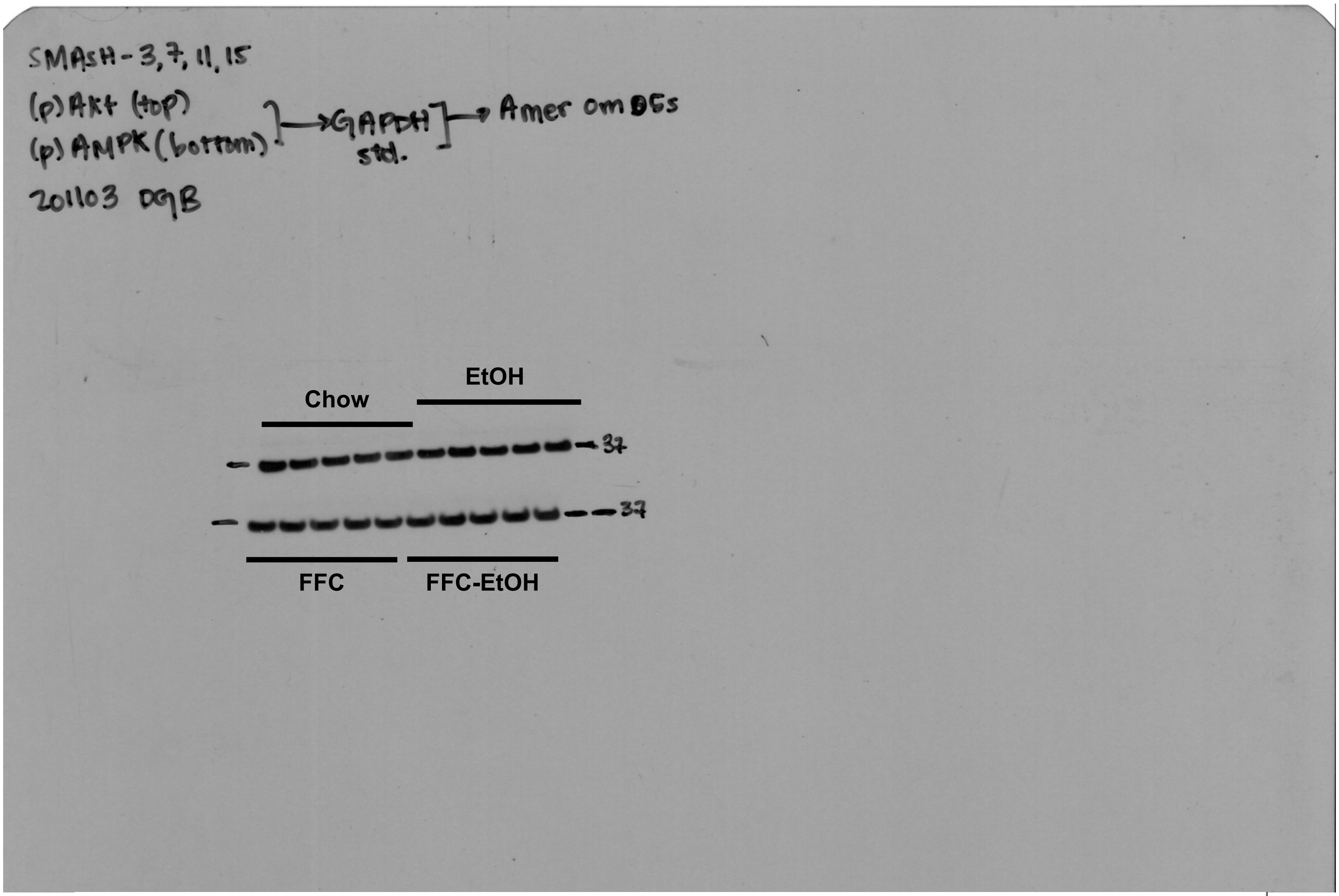
SMASH-3,7,11,15
(p)Akt (top)
(p)AMPK (bottom)
GAPDH std.
201103 DGB
Chow
EtOH
37
37
FFC
FFC-EtOH

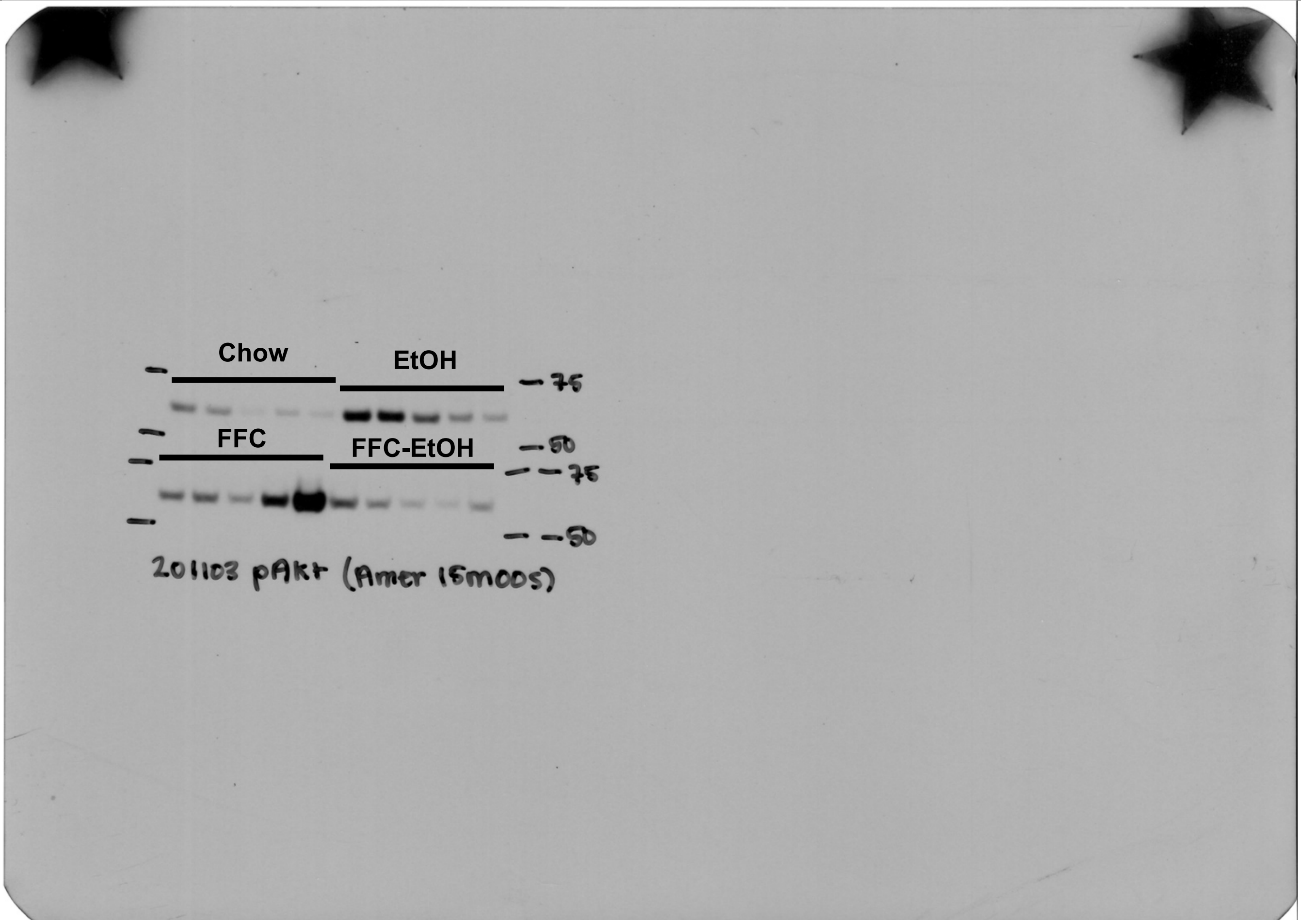
Chow
EtOH
75
FFC
FFC-EtOH
50
75
50
201103 pAkt

Figure 6B

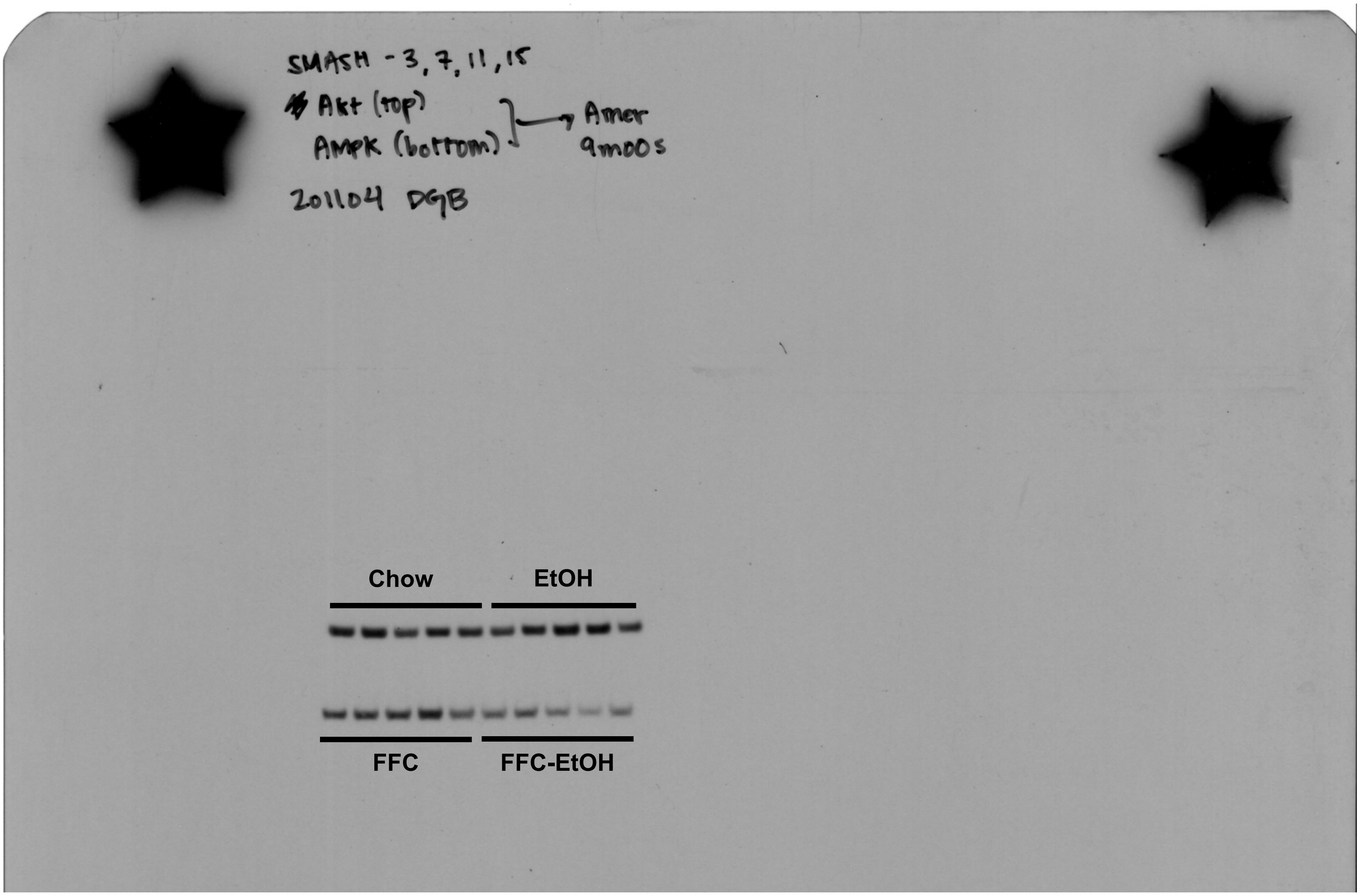
SMASH - 3, 7, 11, 15
Akt (top)
AMPK (bottom)
Amer
9m00s
201104 DGB
Chow
EtOH
FFC
FFC-EtOH

Figure 6C

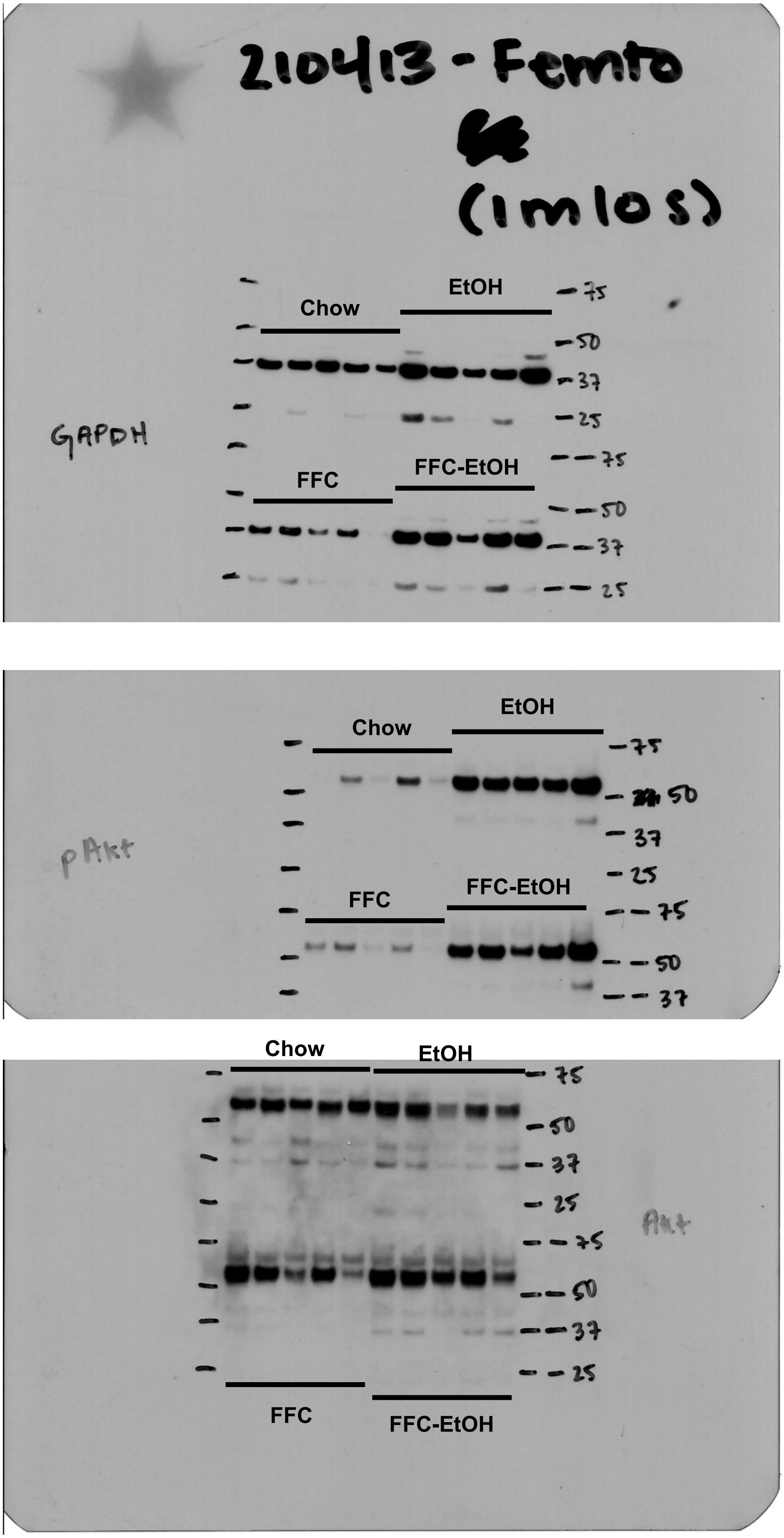
210413 - Femto
(1m10s)
GAPDH
Chow
EtOH
FFC
FFC-EtOH
75
50
37
25
pAkt
Akt

Supplemental Figure 1

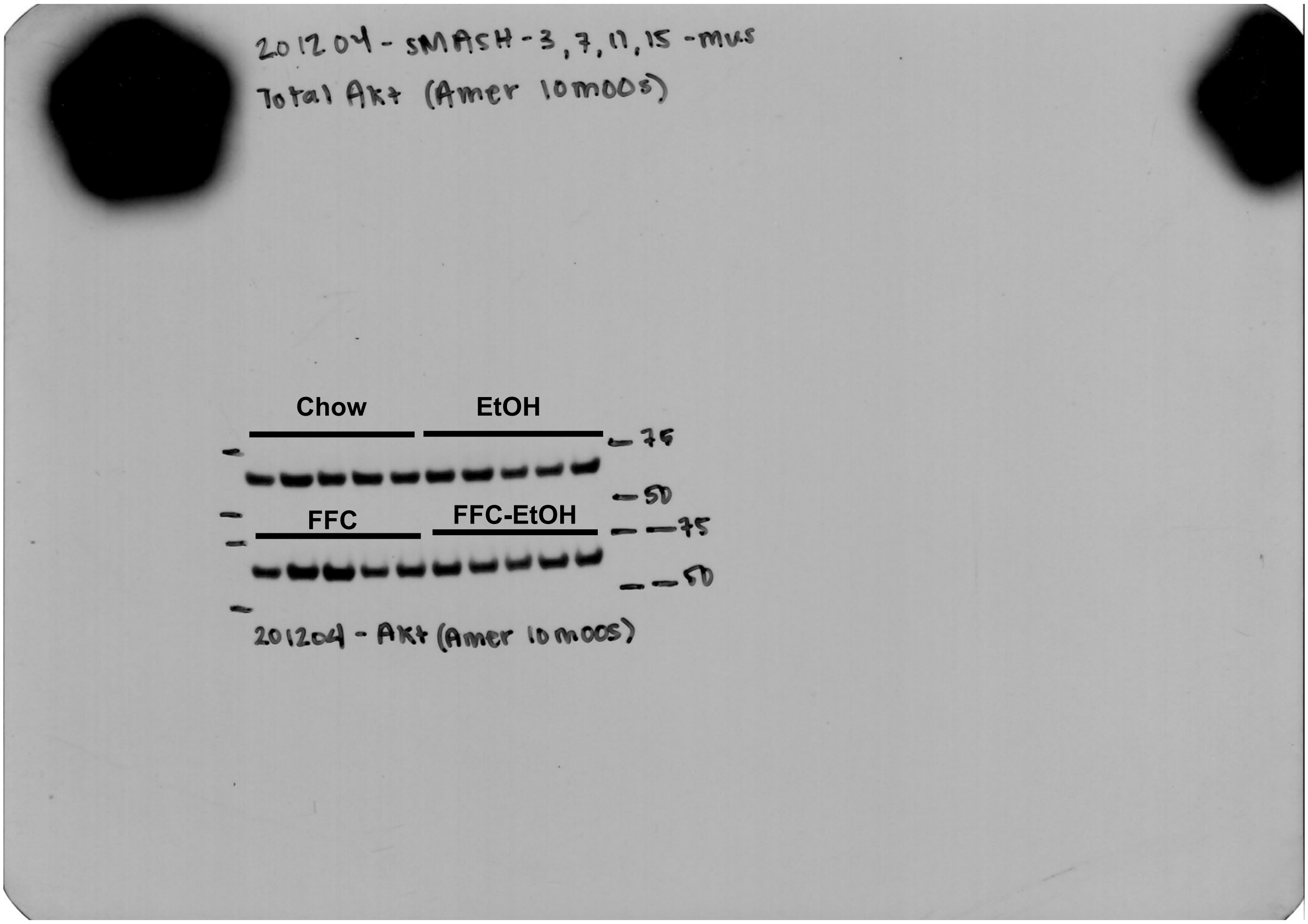

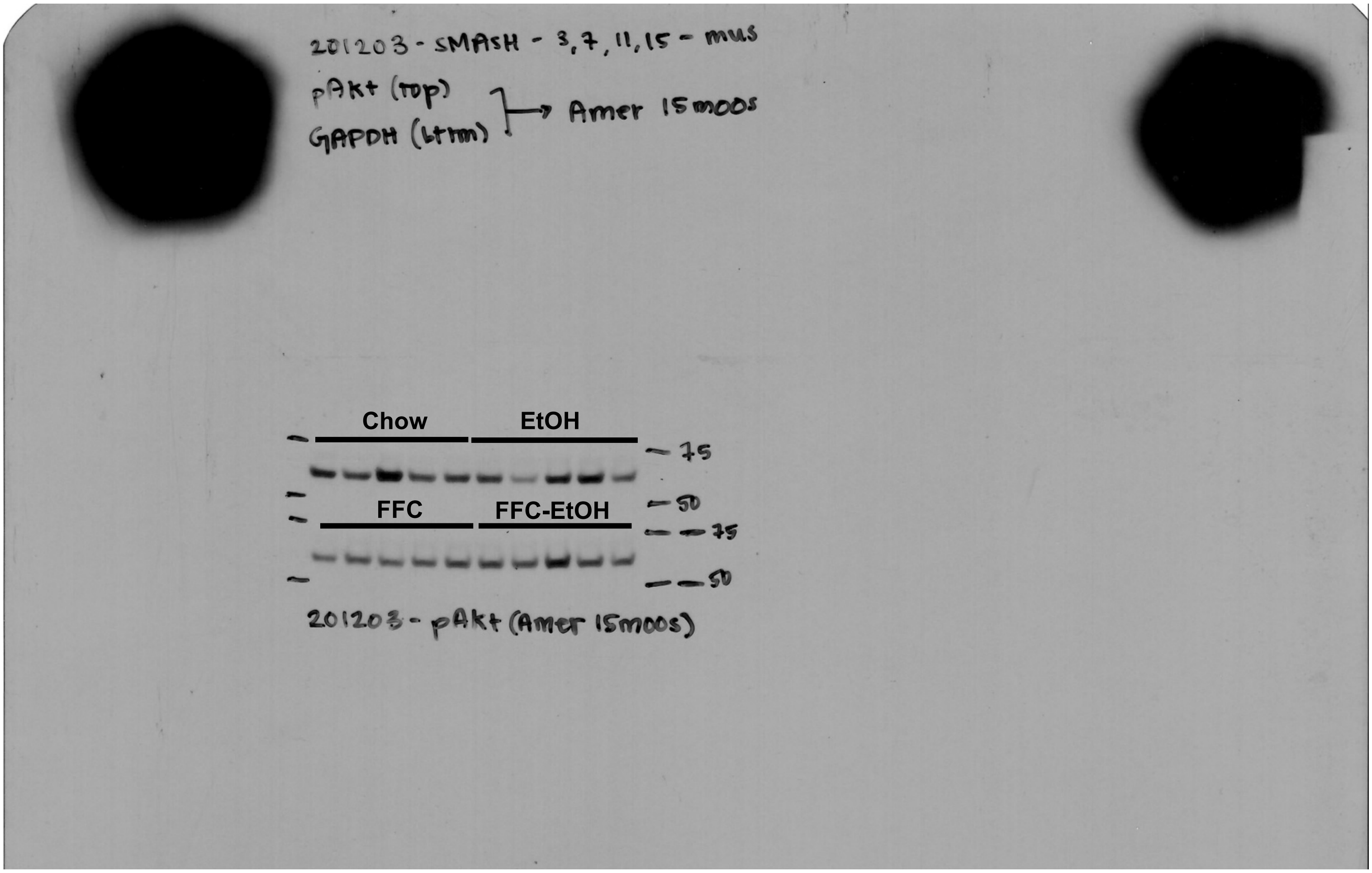

Supplemental Figure 1

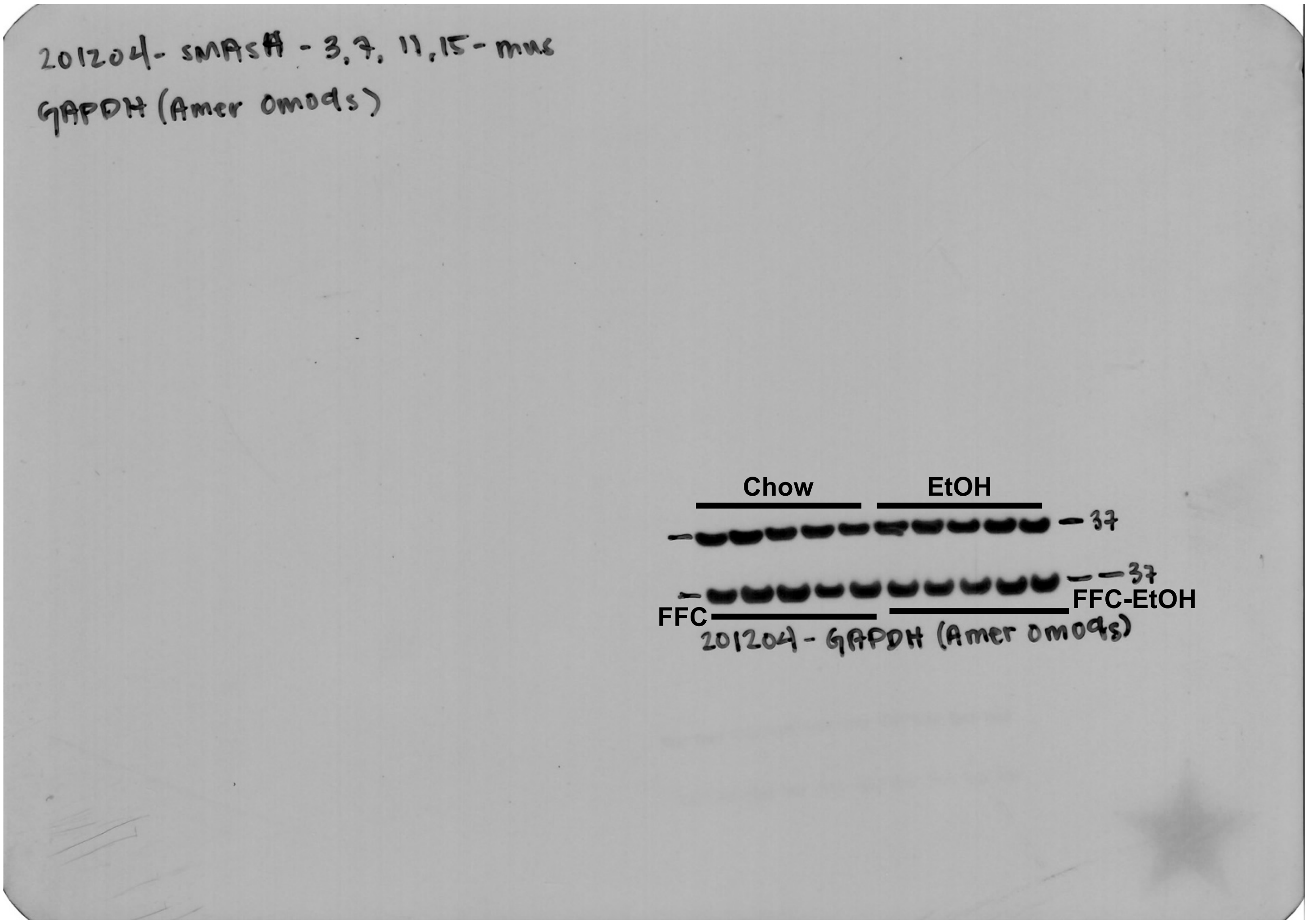

Supplement: S1 Raw images — (PDF) [file pone.0281954.s005.pdf]
